# Coumarin biosynthesis genes are required after foliar pathogen infection for the creation of a microbial soil-borne legacy that primes plants for SA-dependent defenses

**DOI:** 10.1038/s41598-022-26551-x

**Published:** 2022-12-28

**Authors:** Gilles Vismans, Sietske van Bentum, Jelle Spooren, Yang Song, Pim Goossens, Josep Valls, Basten L. Snoek, Benjamin Thiombiano, Mario Schilder, Lemeng Dong, Harro J. Bouwmeester, Pierre Pétriacq, Corné M. J. Pieterse, Peter A. H. M. Bakker, Roeland L. Berendsen

**Affiliations:** 1 Department of Biology, Science4Life, Plant-Microbe Interactions, Institute of Environmental Biology, Utrecht University, 3508 CH Utrecht, the Netherlands; 2 Univ. Bordeaux, INRAE, UMR 1366 OENO - Axe Molécules À Intérêt Biologique, ISVV, 33140 Villenave d’Ornon, France; 3 Department of Biology, Science4, Life Theoretical Biology and Bioinformatics, Institute of Biodynamics and Biocomplexity, Utrecht University, 3508 CH Utrecht, the Netherlands; 4 Plant Hormone Biology Group, Swammerdam Institute for Life Sciences, University of Amsterdam, 1000 BE Amsterdam, the Netherlands; 5 Université de Bordeaux, INRAE, UMR1332 Biologie du Fruit et Pathology, 33882 Villenave d’Ornon, France; 6 Bordeaux Metabolome, MetaboHUB, PHENOME-EMPHASIS, 33140 Villenave d’Ornon, France

**Keywords:** Plant immunity, Plant symbiosis, Secondary metabolism, Microbial communities, Environmental microbiology, Microbiology, Plant sciences

## Abstract

Plants deposit photosynthetically-fixed carbon in the rhizosphere, the thin soil layer directly around the root, thereby creating a hospitable environment for microbes. To manage the inhabitants of this nutrient-rich environment, plant roots exude and dynamically adjust microbe-attracting and -repelling compounds to stimulate specific members of the microbiome. Previously, we demonstrated that foliar infection of *Arabidopsis thaliana* by the biotrophic downy mildew pathogen *Hyaloperonospora arabidopsidis* (*Hpa*) leads to a disease-induced modification of the rhizosphere microbiome. Soil conditioned with *Hpa*-infected plants provided enhanced protection against foliar downy mildew infection in a subsequent population of plants, a phenomenon dubbed the soil-borne legacy (SBL). Here, we show that for the creation of the SBL, plant-produced coumarins play a prominent role as coumarin-deficient *myb72* and *f6’h1* mutants were defective in creating a *Hpa*-induced SBL. Root exudation profiles changed significantly in Col-0 upon foliar *Hpa* infection, and this was accompanied by a compositional shift in the root microbiome that was significantly different from microbial shifts occurring on roots of *Hpa*-infected coumarin-deficient mutants. Our data further show that the *Hpa*-induced SBL primes Col-0 plants growing in SBL-conditioned soil for salicylic acid (SA)-dependent defenses. The SA-signaling mutants *sid2* and *npr1* were unresponsive to the *Hpa*-induced SBL, suggesting that the protective effect of the *Hpa*-induced shift in the root microbiome results from an induced systemic resistance that requires SA-signaling in the plant.

## Introduction

Microbial communities that associate with the plant root, together referred to as the root microbiome, are the focus of numerous studies that explore their potential to enhance plant health and growth^1–4^. Plants sculpt and sustain the root microbiome by depositing large amounts of photosynthetically-fixed carbon in the rhizosphere, creating nutrient-rich conditions for microbial growth and activity^5,6^. With a punishment and reward strategy, plants can modulate rhizosphere microbiome composition by the exudation of both microbe-stimulatory and -repellent metabolites^7–11^. By selectively adjusting the rhizosphere microbiome to favor beneficial microbes, plants can create conditions advantageous to their growth and health.

Previously, it was discovered that plants can dynamically recruit beneficial microbes in response to pathogen attack. Aboveground infection of the model plant *Arabidopsis thaliana* (hereafter: Arabidopsis) with *Hyaloperonospora arabidopsidis*^12^ (hereafter: Hpa) or *Pseudomonas syringae* pv*. tomato*^13^ led to specific changes in the root microbiome. A subsequent population of plants grown in soils thus conditioned with pathogen-infected plants was more resistant to the aboveground attacker. Moreover, the Arabidopsis root microbiome of *Hpa*-infected plants was enriched for a *Xanthomonas,* a *Stenotrophomonas* and a *Microbacterium* species. These bacteria were isolated and cultured, and only when all three were applied as a consortium they consistently induced systemic resistance (ISR) against *Hpa*^12^. Thus, we hypothesize that pathogen infection leads to changes in root exudation profiles that specifically promote beneficial microbes in their root microbiome and in turn trigger ISR to fend off subsequent pathogen attack. By increasing the abundance of these beneficial microbes in the rhizosphere, plants create a soil-borne legacy (SBL) that can protect a next generation of plants growing in that same soil^14^. The dynamic recruitment of beneficial microbes is suggested to be important for the buildup of disease suppressiveness in the field. This is exemplified by a study with sugar beet, in which attack by the soil-borne pathogen *Rhizoctonia solani* led to the activation of a disease-suppressive microbial consortium that protected the plant by antagonizing the pathogen^15^.

It is known that specific beneficial microbes can prime a plant’s immune system and bring about a state of ISR^16,17^. In this primed state of ISR, a plant activates defense responses upon pathogen detection much quicker and stronger, but without the plant growth penalty that is often associated with increased resistance^18–20^. Root colonization by *Pseudomonas simiae* WCS417 (hereafter: WCS417) can elicit ISR in Arabidopsis that is effective against a wide range of pathogens^21–23^. WCS417-mediated ISR is dependent on the transcription factor MYB72 that is expressed in the roots only, but is essential for the onset of ISR systemically^21^. MYB72 is also important in the plant’s iron deficiency response, as it regulates the biosynthesis and secretion of scopoletin^8,24^, a coumarin involved in mobilizing iron from the soil^25^. Moreover, scopoletin has specific antimicrobial activity that inhibits growth of soil-borne pathogens, such as *Fusarium oxysporum* and *Verticillium dahlia,* but to which beneficial *MYB72*-inducing WCS417 bacteria are tolerant^8^. MYB72 thus seems to serve a double role in signaling the onset of ISR as well as shaping the root microbiome. It was hypothesized that MYB72-dependent coumarin secretion is part of a positive feedback loop that stimulates colonization by *MYB72*-inducing and coumarin-tolerant beneficial microbes^8,26–28^, thus providing both enhanced pathogen protection and increased availability of iron in the soil environment.

In this study, we aimed to obtain mechanistic insight into how *Hpa-*infected plants create a SBL and how plants growing in SBL-conditioned soil become protected against *Hpa* infection. We found that the coumarin biosynthesis genes *MYB72* and *F6’H1* are essential for the creation of the SBL, as aboveground *Hpa*-infection in coumarin-deficient mutants *myb72* and *f6’h1* did not result in the creation of an effective SBL. We found that the secretion of a wide range of root metabolites is affected by foliar *Hpa*-infection and identified microbial taxa in the rhizosphere that differently respond to *Hpa*-infection on wild type and the coumarin-deficient mutant plants. Moreover, we found that plants growing on SBL-conditioned soil are primed for enhanced SA-dependent defenses, and that SA signaling mutants do not mount an ISR against *Hpa* on SBL-conditioned soil. Together these data highlight the role of coumarins in the build-up of a foliar pathogen-induced SBL and that this SBL is associated with the recruitment of a disease resistance-inducing microbiome that primes SA-dependent defenses in plants growing in the SBL-conditioned soil.

## Materials and methods

### SBL experiments

In this study, natural soil collected from the Reijerscamp nature reserve in the Netherlands was used^12^. The SBL setup was performed as previously described in detail (Methods S1)^29^. Briefly, approximately 30 Arabidopsis seeds were sown on 60-ml pots filled with soil and placed in a climate-controlled plant growth chamber at 21 °C, 70% relative humidity, 10-h light/14-h dark cycles. Two weeks after sowing, half of the pots were inoculated with a 50 spores/µl suspension of *Hyaloperonospora arabidopsidis* noco2 (*Hpa)* as previously described^30^. Briefly, an airbrush was used to spray spores suspension until tiny droplets started to form on the leaves of the plants, approximately 0.5 ml of spore suspension per pot. The other pots were sprayed with tap water as mock treatment. *Hpa* symptoms were allowed to develop for one week, after which all aboveground material of this so-called “conditioning population” of plants was cut off using a razor blade. A new population of plants was sown and grown as described above. After two weeks of growth of this second plant population, all plants were inoculated with *Hpa* and symptoms were allowed to develop for one week. Disease severity in this so-called SBL “responding population” of plants was determined based on *Hpa* spore densities normalized for plant fresh weight.

Wild-type plants of the Arabidopsis Col-0 accession (N1093; Nottingham Arabidopsis Stock Centre) were used in both the conditioning phase of the experiment (1st population of plants) and in the response phase (2nd population of plants). Depending on the experiment, Col-0 wild-type plants were replaced by mutant *myb72-2,* which is impaired in both rhizobacteria-mediated ISR^21^ and in the biosynthesis and secretion of coumarins^8,24^, the coumarin biosynthesis mutant *f6’h1,* which is impaired in the coumarin biosynthesis enzyme Feruloyl-CoA 6'-Hydroxylase1^31^*,* or by SA signaling mutants *npr1-1*^32^ or *sid2-1*^33^. As controls, pots were left unplanted in either the conditioning phase or the response phase of the experiment. All experiments were performed at least twice with similar results.

### Defense priming assay

To test for priming of plant defenses, 2-week-old plants in the response phase of an SBL experiment were dipped in a 1 mM SA solution supplemented with 0.015% Silwet L-77 or in a mock solution containing 0.015% Silwet L-77. Plants were harvested at 30 min, 4 h, and 6 h after dipping, immediately snap frozen in liquid nitrogen and stored at − 80 °C until further processing. RNA extraction was performed as described by Oñate-Sánchez^34^ with minor modifications. Quantitative real-time PCR (qPCR) analysis of the defense-related marker gene *PATHOGENESIS-RELATED1* (*PR1*) was performed as described by Van Wees et al.^35^.

### Preparation of amplicon sequencing library

For microbiome analysis, the roots from at least ten replicate pots were collected for each treatment at the end of the SBL conditioning phase. DNA was extracted using the DNeasy® Powerlyzer® Powersoil® kit (product #: 12855-100).The *16S rRNA* gene amplicon libraries were constructed using the 16S metagenomic sequencing library protocol for the Illumina MiSeq system^36^ using 16S V3-V4 phasing primers adapted from De Muinck et al.^37^ (Table S1) and the barcoded PCR primers described by Baym and co-workers^38^. The pooled library was sent for sequencing on a 2 × 300 MiSeq at the USEQ sequencing facility (Utrecht University, the Netherlands). The fungal amplicon libraries were constructed in the same way but using primers targeting the internal transcribed spacer (ITS) region 2 of the ribosome encoding genes^39^.

### Amplicon sequence data pre-processing

The 16S and ITS amplicon sequence data was analyzed using Qiime2 version 2019.7^40^ as described in Methods S1. Reads annotated as chloroplast or mitochondria were removed from the data sets and only bacterial amplicon sequence variants (ASVs) with a total relative abundance ≥ 0.01% and detected in at least 10% of the samples were used for further analysis. Fungal ASVs with a total relative abundance of abundance ≥ 0.01% and detection in at least 10% of samples were used for downstream analysis.

### Differential abundance analysis

Five statistical methods were used for differential abundance testing of ASVs: ANCOM-bc, DESeq2, Fisher’s exact test, Simper analysis and Spearman rank correlations^41–43^. ASVs were considered differentially abundant if the FDR-adjusted *P* ≤ 0.05 as generated by ANCOM-bc, DESeq2, and Spearman rank correlations. For Fisher’s exact test and Simper analysis we used unadjusted *P* ≤ 0.05 as the threshold for significance. Sparse ASVs denoted as structural zeroes by ANCOM-bc were ignored in downstream analysis unless also detected by at least one other statistical method.

### Software used

**Figure 1:**
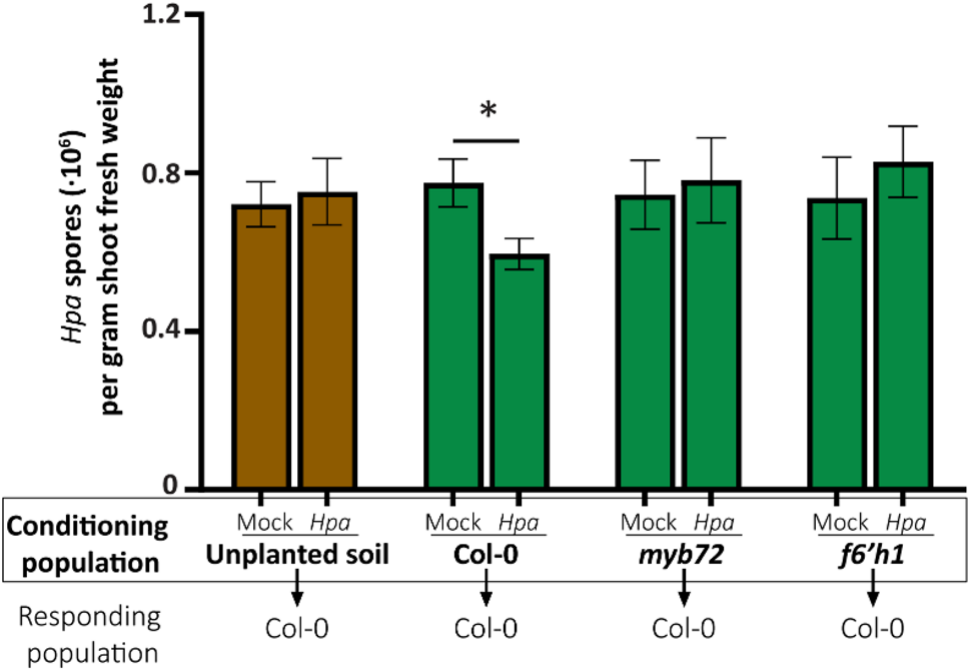
Coumarin biosynthesis is essential for the creation of a *Hpa-*induced SBL that enhances resistance against downy mildew in a responding population of plants. Green bar graphs represent the disease severity of SBL-responding populations of Col-0 plants growing on natural Reijerscamp soil pre-conditioned with mock-treated or *Hpa-*inoculated Col-0, *myb72*, or *f6’h1* seedlings (Experiment 1). Brown bar graphs represent the disease severity of responding populations of Col-0 plants growing in control pots that during the conditioning phase remained unplanted and were mock- or *Hpa*-inoculated directly on the soil. Bars depict the average *Hpa* spore production per gram of shoot fresh weight (n = 10). Error bars depict standard error of the mean. Asterisk denotes a significant difference (*P* = 0.01 in Students *t*-test).

**Figure 2:**
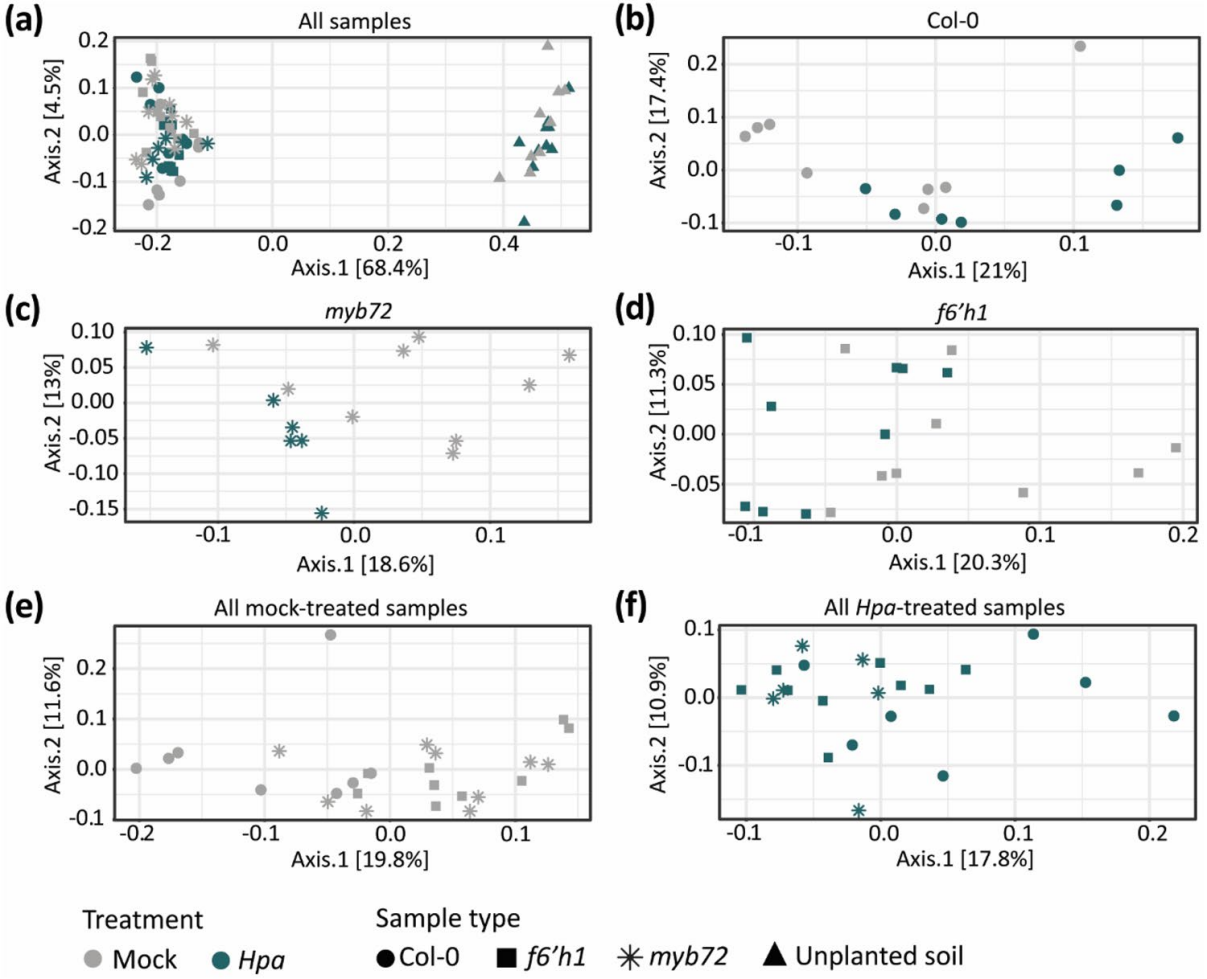
Effect of foliar *Hpa* infection on root bacterial communities. (**a**) Principal coordinate analysis (PCoA) of bacterial communities in unplanted soil (filled triangle) and the rhizospheres of 3-week-old seedlings of Col-0 (filled circle), *myb72* (⁎) and *f6’h1* (filled rectangle). Samples were taken one week after either mock treatment (grey symbols) or inoculation with *Hpa* (blue-green symbols). (**b**) PCoA of only the Col-0 samples. (**c**) PCoA of only the *myb72 samples.* (**d**) PCoA of only the *f6’h1* samples. (**e**) PCoA of root bacterial communities of mock-treated Col-0 (filled circle), *myb72* (⁎) and *f6’h1* (filled rectangle) plants. (**f**) PCoA of root bacterial communities of *Hpa*-infected Col-0 (filled circle), *myb72* (⁎) and *f6’h1* (filled rectangle) plants. Statistical tests on differences between microbial communities in the different genotypes and treatments is given in Table S2.

**Figure 3:**
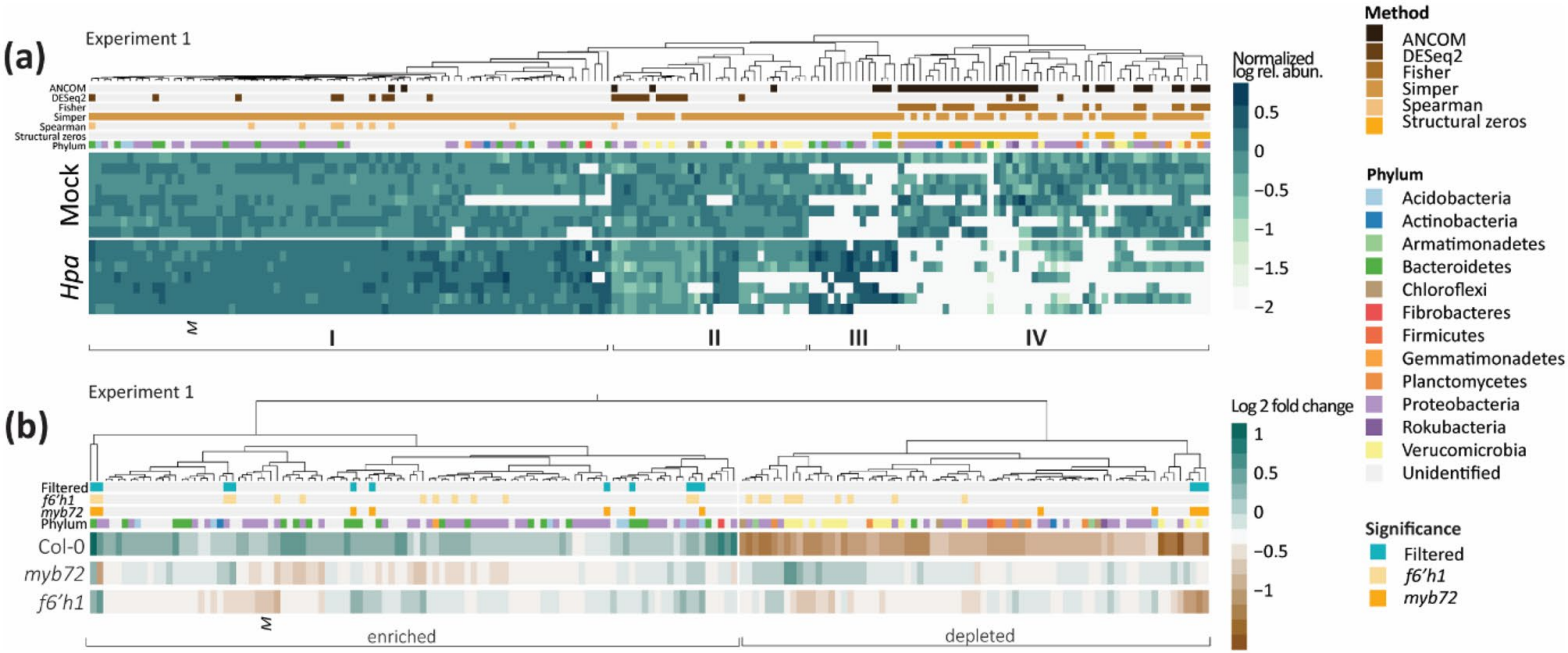
Differentially abundant bacterial ASVs in the rhizosphere of *Hpa*- and mock-treated Col-0, *myb72* and *f6’h1* plants in Experiment 1. (**a**) Heatmap showing the log-transformed relative abundance of 176 ASVs that are differentially abundant between mock- and *Hpa*-treated Col-0 plants. Relative abundances were normalized by the average relative abundance of that ASV across all mock-treated plant samples. Colors above heatmap indicate phylum annotation and statistical method(s) by which each differentially abundant ASV was identified. I-IV indicate clusters of ASVs with similar differential abundance patterns between *Hpa*- and mock-treated plants. (**b**) Fold change of the 176 *Hpa*-responding ASVs between Col-0, *myb72* and *f6’h1* plants. Shrunk fold change in relative abundance between mock- and *Hpa*-treated plants is shown as calculated with DESeq2. Colors above the heatmap indicate phylum annotation and whether an ASV was also differentially abundant between mock- and *Hpa*-treated *myb72* and *f6’h1* plants. “Filtered” ASVs were differentially abundant and similarly enriched or depleted on Col-0 as well as on at least one plant mutant genotype. Enriched and depleted ASVs are highlighted as clusters with positive or negative log2 fold change in Col-0, respectively. In both heatmaps, *M* indicates *Massilia* ASV 09023.

**Figure 4:**
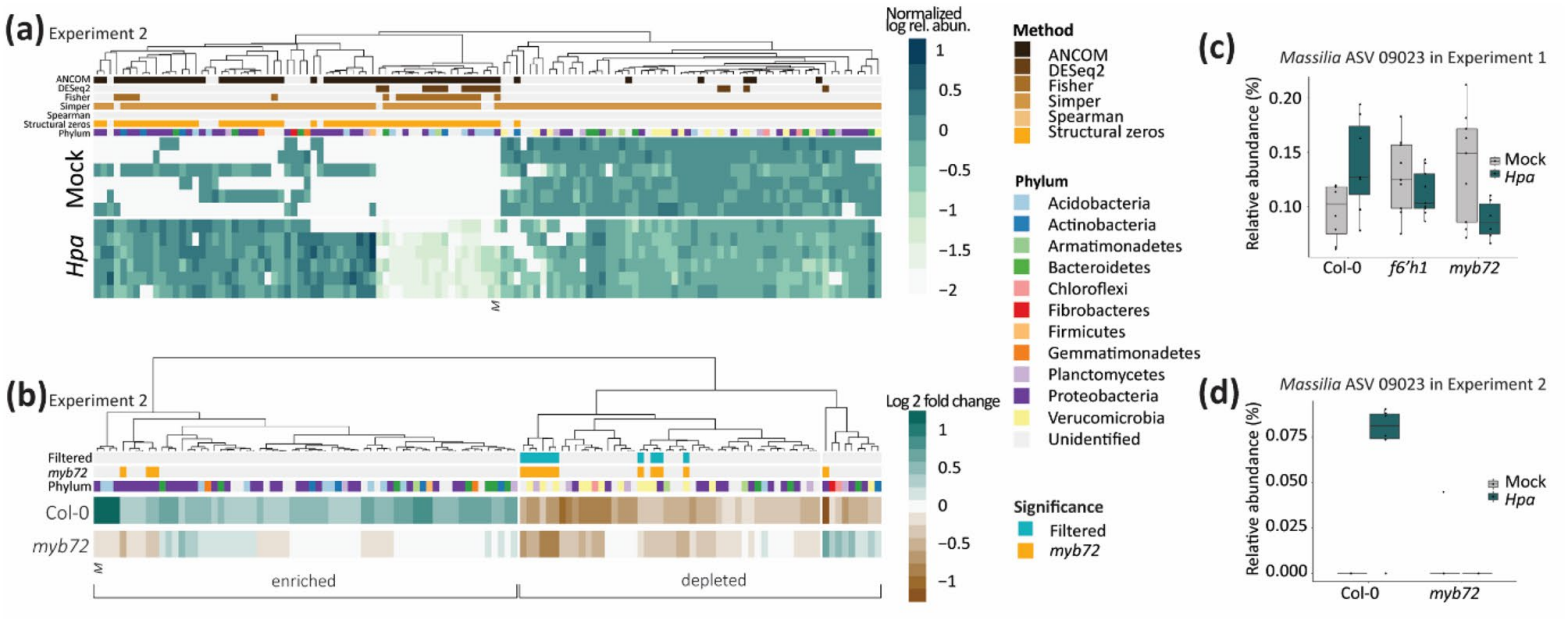
Differentially abundant bacterial ASVs in the rhizosphere of *Hpa*- and mock-treated Col-0 and *myb72* plants in Experiment 2. (**a**) Heatmap showing the log-transformed relative abundance of 120 ASVs that are differentially abundant between mock- and *Hpa*-treated Col-0 plants in Experiment 2. Relative abundances were normalized by the average relative abundance of that ASV across all mock-treated plant samples. Colors above heatmap indicate phylum annotation and statistical method(s) by which each differentially abundant ASV was identified. (**b**) Fold change of the 120 *Hpa*-responding ASVs between Col-0 and *myb72* plants in Experiment 2. Shrunk fold change in relative abundance between mock- and *Hpa*-treated plants is shown as calculated with DESeq2. Colors above the heatmap indicate phylum annotation and whether an ASV was also differentially abundant between mock- and *Hpa*-treated *myb72* plants. Filtered ASVs were differentially abundant and similarly enriched or depleted on Col-0 and *myb72* plants. Enriched and depleted ASVs are highlighted as clusters with positive or negative log2 fold change in Col-0, respectively. In both heatmaps, *M* indicates *Massilia* ASV 09023. (**c**) Relative abundance of *Massilia* ASV 09023 on plants in Experiment 1. (**d**) Relative abundance of *Massilia* ASV 09023 on plants in Experiment 2.

**Figure 5:**
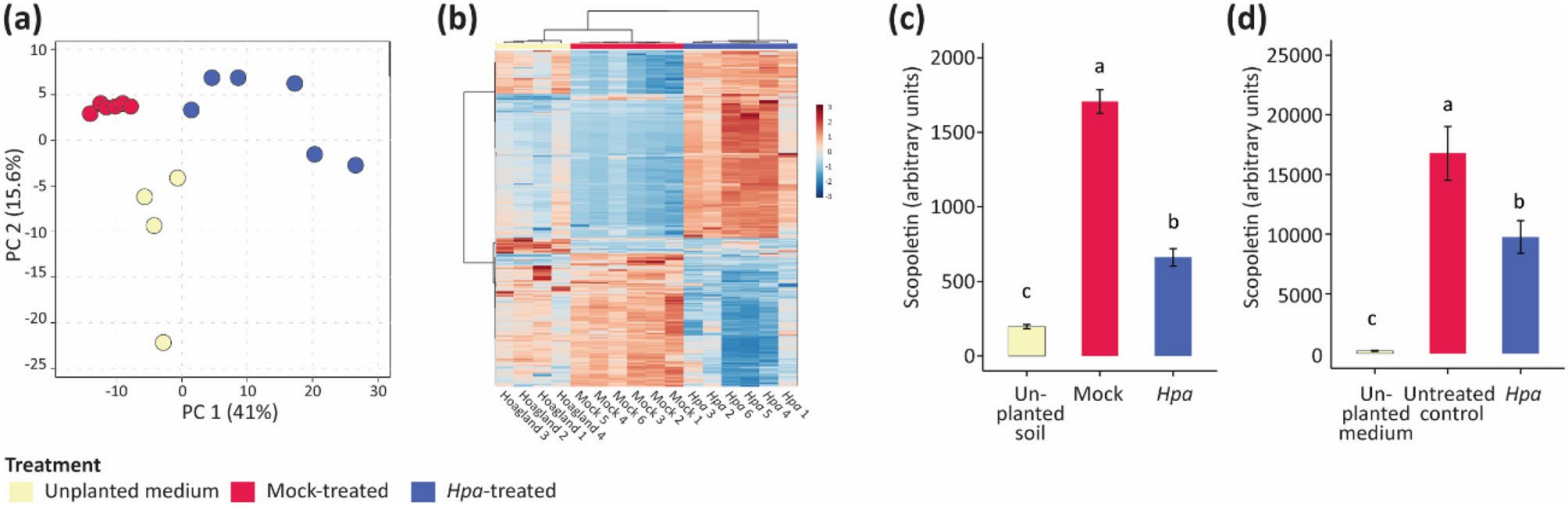
Root exudate profile of Col-0 is affected by foliar *Hpa* infection. (**a**) Principle component analysis (PCA) of 923 metabolic profiles (see Data S3) of sterile HG solution without plants (yellow symbols) or with root exudates of Col-0 plants that were either mock-inoculated (red symbols) or of which the leaves were inoculated with *Hpa* (blue symbols). (**b**) Clustering analysis with heatmap (Pearson’s correlation with Ward clustering) of 459 metabolite markers with significantly different detected levels between the treatments (ANOVA*, P* < 0.01, FDR). Heatmap depicts normalized intensities (blue: depleted, red: accumulated). (**c**) Quantification of scopoletin in root exudates of mock-inoculated or *Hpa*-infected plants growing in natural Reijerscamp soil. As a control, scopoletin levels were measured in unplanted soil. Bars represent the average of 5 samples. (**d**) Quantification of scopoletin in root exudates of mock-inoculated or *Hpa*-infected plants growing in gnotobiotic conditions on agar-solidified HG medium. As a control, scopoletin was measured in unplanted agar plates. Bars represent the average of 5 samples. Letters indicate significant differences (*P* < 0.05 in ANOVA with Tukey’s posthoc test) between treatments. Error bars show standard error.

All statistical analyses on the 16S amplicon sequencing data were performed in RStudio version 3.5.0 (data in Figs. 1 and 2) and version 3.6.1 (Figs. 3, 4 and 5). Figures were created in RStudio version 3.6.1 using phyloseq^44^, ggplot2^45^, tidyheatmap (https://github.com/jbengler/tidyheatmap). PRISM8 Graphpad and Adobe Illustrator CC 2017 and 2022 were used to prepare graphs and figures.

### Untargeted metabolomics

Sterile root exudates secreted by Arabidopsis roots in response to foliar *Hpa* inoculation were collected as described by Song et al. (summarized in Methods S1)^46^. Briefly, plants were sown on seed holders filled with agar-solidified 1/10 strength Hoagland (HG) solution. Roots of the germinating seedlings penetrated the agar to grow into vials with full strength HG solution and grown in controlled-climate conditions and kept sterile in Eco2boxes (Eco2 NV). After 19 days, leaves of half of the plants were carefully inoculated with an *Hpa* spore suspension. Five days post-inoculation, plants were removed from the HG medium and the medium was checked for sterility. Contaminated root exudates were discarded and sterile root exudates were lyophilized. Untargeted metabolomics was conducted on exudates using an ACQUITY ultra high performance liquid chromatography (UHPLC) coupled to a SYNAPT G2Si quadrupole time-of-flight (Q-TOF) mass spectrometer equipped with an electrospray (ESI) source (Waters) as previously described^47^ and summarized in Methods S1. Raw LCMS data were processed following the DIA MS2 deconvolution method using MS-DIAL software v. 4.7^48^ and exploiting the online library MSMS-Public-Neg-VS15.msp (36,848 records). Putative annotation of differentially expressed metabolites resulted from MS-DIAL screening of the MS1 detected exact HR *m/z* and MS2 fragmentation patterns against multiple online databases (http://prime.psc.riken.jp/compms/msdial/main.html#MSP).^48^ After data-cleaning (blank check, SN > 10, CV QC < 30%), 923 metabolomic variables were retained for further chemometrics. The final dataset was normalized (median normalization, cube-root transformation and Pareto scaling) using MetaboAnalyst v 5.0^49^ prior to multivariate statistical analyses. See Methods S1 for further details.

### Collection of *Hpa*-responsive root exudates from soil

A population of Arabidopsis Col-0 plants was cultivated in the SBL experimental setup. Two weeks after sowing, half of the pots were inoculated with an *Hpa* spore suspension (50 spores/µl water) or with water. Seven days post-inoculation (dpi), approximately 65 ml of 5% ethanol was poured on top of the pots and allowed to flow through the soil. Following saturation of the soil, 50 ml of soil effluent was caught in 50-ml Falcon tubes and immediately frozen at − 20 °C. Samples were lyophilized for further processing.

### Collection of *Hpa*-responsive root exudates from agar-solidified medium

Arabidopsis seeds were surface-sterilized and sown on agar-solidified Murashige and Skoog medium supplemented with 0.5% sucrose. After 4 days of stratification, plates were transferred to a climate chamber (21 °C, 70%, relative humidity, 10 h light/14 h dark, light intensity 100 μmol m^−2^ s^−1^). Twelve-day old seedlings were transplanted to agar-solidified HG medium with 5 seedlings per plate. On the same day, plants were inoculated with *Hpa* spores from a gnotobiotic laboratory-maintained *Hpa* noco2 culture (free of other microbes) by gently touching leaves from the infected plants against leaves of the healthy plants. Healthy plants that remained untreated and plates containing HG agar medium without any plants (empty control) were taken along as well. Plates were placed in sealed trays to ensure high humidity and stored in a climate chamber set at conditions favorable for infection (16 °C, 70% relative humidity, 9 h light/15 h dark, light intensity 100 μmol m^−2^ s^−1^). Seven days post-inoculation, rectangular pieces of agar medium were cut from the area occupied by the root elongation zone of plants that were visibly sporulating. Agar pieces were collected and pooled per plate as one biological replicate. For the empty control, agar samples of similar weight were taken from plates without plants. Samples were snap frozen in liquid nitrogen and stored at − 80 °C. Samples were lyophilized prior to further processing.

### UHPLC-MS/MS analysis of coumarins

For detection of coumarins in root exudates, lyophilized samples were analyzed on a 1290 Infinity II UHPLC system equipped with an InfinityLab Poroshell 120 PFP, 2.1 × 100 (P/N: 695675-408) in combination with a Poroshell 120 PFP, with a 2.1 mm HPLC guard column and a 1.9 µm LC column (P/N: 821725-942; Agilent Technologies) coupled to an Agilent Technologies 6470A Tandem Quadrupole Mass Spectrometer (TQ-MS–MS). Coumarins were subsequently analyzed using MS with an ESI source in positive ion mode. Further details are described in Methods S1.

### Ethics approval and consent to participate

The experimental research on plants in this study complies with relevant institutional, national, and international guidelines and legislation.

## Results

### MYB72 and coumarin biosynthesis are required for the creation of the soil-borne legacy mediated by *Hpa*-infected Arabidopsis

MYB72 regulates the production and secretion of coumarins, most notably scopoletin. Coumarins are secreted by the roots, have antimicrobial activity and can shape root microbiome assembly^8,50^. We therefore hypothesized that MYB72 and coumarin biosynthesis may play a role in the creation of the *Hpa-*induced SBL. This hypothesis was tested by preconditioning Reijerscamp soil with either wild-type Col-0, or the coumarin biosynthesis mutants *myb72* or *f6′h1* that were mock- or *Hpa*-inoculated. Moreover, we included non-planted, soil-filled pots that were sprayed with water or *Hpa* spores during the conditioning phase to investigate the possibility that the SBL was created via the *Hpa* inoculum. After removal of the first “conditioning population” of plants, a “responding population” of Col-0 plants was sown and grown on all pre-conditioned soils and inoculated with *Hpa*. One week post-inoculation, a significant reduction in *Hpa* spore production was observed for Col-0 plants growing on soil pre-conditioned by *Hpa-*infected Col-0 plants (Fig. 1), confirming previous findings (Berendsen et al.^12^). Interestingly, pre-conditioning of soil by the coumarin biosynthesis mutants *myb72* and *f6’h1* did not significantly affect *Hpa* resistance in the responding population of Col-0 plants (Fig. 1). Pre-conditioning of unplanted soil with *Hpa* inoculum had also no effect on *Hpa* resistance in Col-0 plants. We repeated this experiment with similar results (Fig. S1). Our results indicate that coumarin production is a pre-requisite for the creation of the SBL mediated by *Hpa-*infected plants.

### *Hpa* infection results in significant changes in the root microbiome

To investigate the effect of *Hpa* infection on root microbiome assembly, we analyzed the composition of the root microbiomes of the healthy and *Hpa-*infected Col-0, *myb72,* and *f6’h1* plants from the experiment described above (Experiment 1; Fig. 1). To this end, we sequenced 16S and ITS amplicons derived from roots of plants and of unplanted soil sampled at the moment the conditioning population of plants was removed. After filtering, the 16S data for this experiment comprised 67 samples with on average 97,108 reads per sample and a total of 1866 unique ASVs (Data S1). Principal coordinate analysis (PCoA) of these samples revealed that the root microbiome samples of Col-0, *f6’h1* and *myb72* plants clustered away from the unplanted soil samples, highlighting a strong rhizosphere effect for the bacterial communities (Fig. 2a, Table S2). Whereas the application of an *Hpa* spore suspension on unplanted soils did not result in changes in the soil microbiome, aboveground *Hpa* infection of plants led to a statistically significant shift (adjusted *P* < 0.05 in PERMANOVA) in the bacterial communities on the roots of Col-0 plants, as well as of the coumarin biosynthesis mutants *myb72* and *f6’h1* (Fig. 2b–d, Table S2). This shift constituted a change in the composition of the root bacterial communities, whereas the α-diversity of the communities was not affected (Fig. S2). Moreover, the bacterial communities on the roots of the mock-treated mutant plants were significantly different from those on the roots of mock-treated wild-type plants, whereas the bacterial communities on the roots of both mock-treated coumarin-deficient mutants did not differ from each other (Fig. 2e, Table S2). Also after *Hpa*-inoculation, the root bacterial communities on mutant plants were different from those on Col-0 plants (Fig. 2f), although the difference between infected *myb72 and* Col-0 plants was not significant at the *P* = 0.05 level (*P* = 0.081, Table S2).

For the fungal ITS amplicon data, filtering resulted in a dataset of 74 samples with a total of 6,700,000 reads and 167 unique fungal ASVs. Also the fungal communities showed a clear rhizosphere effect as the roots of healthy and *Hpa*-inoculated Col-0, *myb72* and *f6’h1* plants were significantly different from the unplanted soil samples (adjusted *P* < 0.05 in PERMANOVA; Fig. S3, Table S2). In contrast to the bacterial microbiota (Table S2), root fungal microbiota were not significantly affected by foliar *Hpa* infection (Fig. S3, Table S3). This indicates that the root fungal microbiome is unlikely to play a role in the *Hpa* infection-mediated SBL and, for the further analyses in this study, we focused on the *Hpa*-induced changes in the bacterial root microbiome.

### A large cluster of ASVs is significantly enriched on roots of *Hpa*-infected Col-0 plants

To dig deeper in the *Hpa* infection-mediated changes in the root microbiome, we analyzed the differential abundance of individual ASVs between healthy and *Hpa*-infected plants at the end of the conditioning phase of the experiment presented in Fig. 1. Several methods of differential abundance testing are well-accepted in literature and each of these methods has strengths and weaknesses^51,52^. For example, DESeq2^43^ can detect small but consistent differences in ASV abundance, but in our experience is unlikely to detect ASVs that are sparsely present in at least one of two treatments. To make optimal use of the available methods of differential abundance testing, we selected ANCOM-bc, DESeq2, Fisher’s exact test, Simper, and Spearman’s correlation as complementary methods to analyze our data^41–43^. We identified 176 differentially abundant ASVs between roots of mock- and *Hpa*-inoculated Col-0 plants that were detected by at least 1 of these 5 methods (Fig. 3a). Among these 176 ASVs, we found a cluster of ASVs (Fig. 3a; cluster I) that occur in most samples but are enriched on the roots of *Hpa-*infected plants, and a smaller cluster of ASVs (cluster II) with frequent occurrence that are depleted on the roots of *Hpa-*infected plants. Moreover, another two clusters of ASVs are sparse in one of the two treatments, but occur frequently and abundantly in the other treatment. Also among these more sparse ASVs we identify ASVs that are enriched on the roots of *Hpa*-infected plants (cluster III) as well as ASVs that are depleted on roots of *Hpa-*infected plants (cluster IV).

In the conditioning phase, only *Hpa-*infected Col-0 plants, not the coumarin-deficient mutants *myb72* and *f6’h1*, created a SBL that increased *Hpa* resistance in the second, responding population of plants (Fig. 1). Thus, to identify microbiota that are associated with this increased resistance, we subsequently examined the response of rhizosphere-associated bacterial taxa to foliar *Hpa* infection on roots of *myb72* and *f6’h1* plants. Of the 176 ASVs responding to *Hpa* infection on roots of Col-0 plants, 14 ASVs responded significantly and were similarly enriched or depleted on at least one of the coumarin-deficient mutants (Fig. 3b). Hence, it is unlikely that the microbes corresponding to these ASVs are part of the disease-induced SBL on Col-0 plants that induces resistance against *Hpa* in a subsequent population of plants. The remaining 162 ASVs differentially abundant on Col-0 plants were either not significantly responsive to *Hpa* infection or inversely responsive on either *myb72*, *f6’h1* or both mutant genotypes. From these 162 ASVs, 100 ASVs were enriched on the roots of *Hpa*-infected Col-0 plants (Fig. 3b), thus potentially responsible for the increased *Hpa* resistance in the response population of Col-0 plants. These 100 ASVs represented 12 different bacterial phyla, of which most belonged to the phylum Proteobacteria. Together, these data show that a group of phylogenetically diverse microbes increased in abundance on the roots of foliar-infected Col-0 plants, while their abundances remained stable or depleted on roots of *Hpa-*infected coumarin biosynthesis mutants.

### Consistent enrichment of ASVs across two experiments

The SBL is reproducibly generated by *Hpa*-infected plants in independent experiments, and thus it was further investigated if there is consistency in the identity of microbes that are recruited by the roots of *Hpa*-infected plants. We analyzed the root microbiome of an independent experiment (Experiment 2) that included both Col-0 and *myb72* plants in the conditioning phase. This experiment was carried out in soil from the same Reijerscamp field but was collected several months after collection of the soil for the first experiment (October 2018 vs April 2019). After quality control and filtering, the 16S data comprised 32 samples with on average 54,842 reads per sample comprising a total of 2003 unique ASVs over all samples. In this second experiment, again a significant shift in the bacterial root microbiome was detected in response to foliar *Hpa* infection in both Col-0 and *myb72* plants (*P* = 0.013; Fig. S4, Table S4, Data S2). In this experiment, the bacterial root communities of healthy *myb72* plants were not significantly different from those on healthy Col-0 plants (*P* = 0.098), whereas the differences in bacterial root communities between *Hpa*-infected Col-0 and *myb72* plants were significant (P = 0.004).

In this second experiment, 120 ASVs were differentially abundant between the roots of *Hpa-*infected and healthy Col-0 plants (Fig. 4a). Of these, 110 ASVs did not respond similarly to *Hpa* infection in *myb72* mutant plants. Thirteen of the 110 MYB72-dependent ASVs were significantly affected by *Hpa* infection in both the first and the second experiment. One of the 13 steadily affected ASVs on roots of *Hpa*-infected Col-0 plants was consistently enriched, while 7 ASVs were consistently depleted. The remaining 5 ASVs responded in varying directions between the two experiments (Fig. S5). The only ASV that was consistently enriched on roots of *Hpa*-infected Col-0 plants was annotated as a *Massilia* spp. This *Massilia* ASV 09023 was present in almost all root microbiome samples of the first experiment (Fig. 3a) and increased in abundance on Col-0 roots following *Hpa* infection, whereas it was depleted on the roots of *Hpa*-infected coumarin-deficient mutant plants (Figs. 3b, 4c). In the Experiment 2, this ASV was detected on the roots of most of the *Hpa-*infected Col-0 plants (5 out of 6) but virtually absent from all *myb72* and mock-treated wild-type plants (Fig. 4a,d). Together, the data of this second experiment confirms that there is a phylogenetically diverse set of rhizosphere microbes that changes in abundance in response to foliar *Hpa* infection in a coumarin-dependent manner. Within the rhizosphere microbial community responding to foliar *Hpa* infection, a *Massilia* spp. was consistently enriched.

### Root exudate composition and exuded coumarins are significantly affected by foliar *Hpa*-infection

In contrast to Col-0, mutant *myb72* and *f6’h1* plants cannot create an *Hpa-*induced SBL that increases resistance in a response population of Col-0 plants (Fig. 1), suggesting that root-secreted coumarins are required for the creation of an effective SBL. To examine how *Hpa*-infection affects root exudation, we first analyzed sterile root exudates of healthy and *Hpa*-infected Col-0 plants with an untargeted metabolomics approach. UPLC-QTOF-MS detected 923 metabolite features that were visualized by PCA, revealing that *Hpa* infection clearly impacted root exudate patterns (Fig. 5a). Clustering analysis of 459 significant metabolite features (ANOVA, *P* < 0.01, FDR) further confirmed that root exudates of *Hpa*-infected plants differ significantly from those of healthy plants (Fig. 5b). Based on accurate *m/z* and fragmentation patterns, we were able to putatively annotate metabolite features that were significantly enriched in the root exudates of diseased plants, in which especially compounds that belong to phenolic biosynthesis pathways (e.g. the shikimate pathway) and derivatives of amino and organic acids accumulated (Data S3). Conversely, phenolic glycosides, coumaric acids and azelaic acid were depleted in the exudates of infected plants. Using this untargeted approach, we were unable to detect the known MYB72- and F6’H1-dependent coumarins esculetin, fraxetin and scopoletin. Therefore, we re-analyzed the sterile root exudates by HPLC with a SB-C18 column using commercially available standards of esculetin, fraxetin and scopoletin to target our methodology. However, we again could not detect these specific coumarins. This could mean that they are not produced under these circumstances, or that this method is not sensitive enough for their detection of these coumarins.

Next we used a coumarin-targeted UHPLC-TQ-MS–MS method to detect whether soil-grown Col-0 plants produce coumarins in response to *Hpa* infection. To this end, Col-0 plants were cultivated in natural Reijerscamp soil and inoculated with *Hpa* similarly as for a SBL assay. Seven days post inoculation, the pots were flushed with 5% ethanol after which the root exudates were analyzed for coumarin accumulation. We found that scopoletin accumulated in the root exudates, but not fraxetin and esculetin. Remarkably, we detected significantly more scopoletin in the root exudates of mock-inoculated plant than in that of *Hpa*-infected plants (Fig. 5c). We hypothesized that scopoletin may be more rapidly metabolized by the microbiome that is recruited by plants in response to foliar *Hpa* infection. We therefore assessed the levels of scopoletin secreted by plants growing on agar-solidified medium that were inoculated with monoxenic *Hpa* spores (Fig. 5d). Again significantly lower levels of scopoletin were detected in the substrate surrounding the roots of *Hpa-*inoculated plants than in that of healthy control plants. These results suggest that foliar *Hpa* infection results in less secretion of scopoletin by the roots. Together our data show that plants respond to foliar infection by altering their root exudation profiles. This response affects a large number of metabolites and while putative phenolics and derivatives of amino and organic acids accumulate around the roots of infected plants they secrete significantly less of the coumarin scopoletin.

### Perception of the soil-borne legacy of *Hpa*-infected Arabidopsis depends on SA and primes foliar defenses

**Figure 6:**
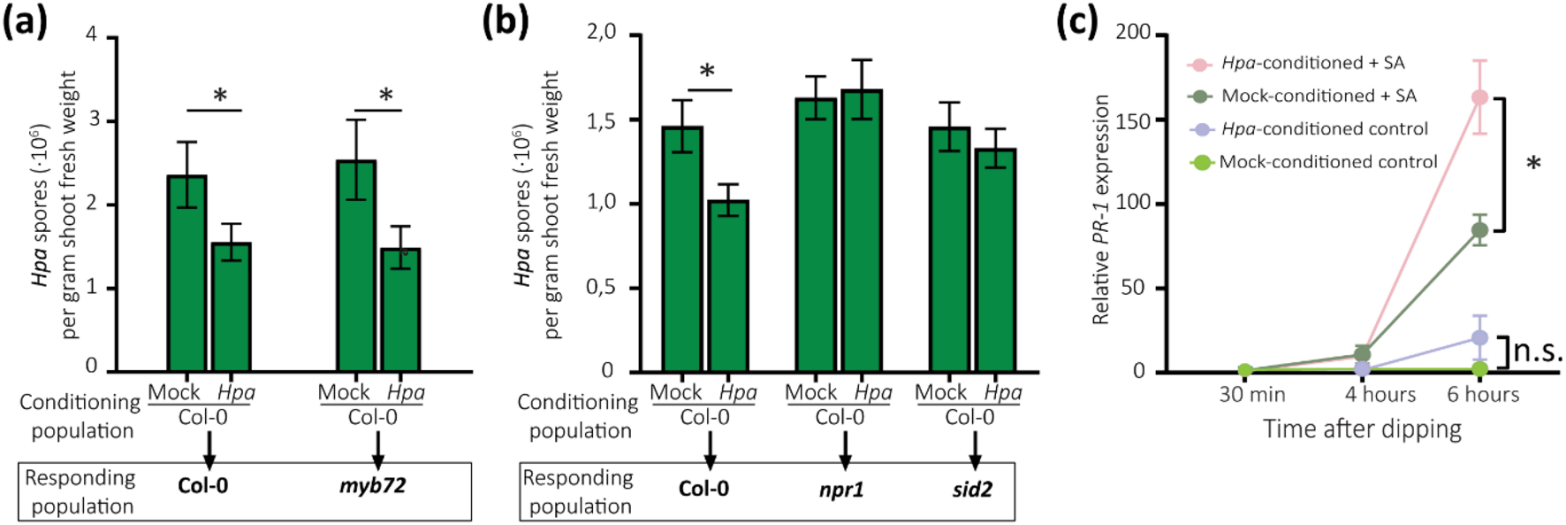
Perception of the *Hpa-*induced SBL is SA dependent and leads to priming of SA-dependent defense responses. (**a**) Disease severity of *Hpa*-inoculated Col-0 and *myb72* plants (Responding population) growing in the on soil pre-conditioned with healthy (Mock) or *Hpa-*infected Col-0 plants. Bars depict the average spore production per gram of shoot fresh weight of 9–10 replicates. Error bars depict standard error of the mean. Asterisk denotes a significant difference (*P* < 0.05 in Students *t*-test). (**b**) Disease severity of *Hpa*-inoculated Col-0, *npr1* and *sid2* plants (Responding population) growing on soil pre-conditioned by healthy (Mock) or *Hpa-*infected Col-0 plants. Bars depict the average spore production per gram of shoot fresh weight of 20 replicates with values based on 2 independent experiments. Error bars depict standard error of the mean. Asterisk indicates a significant difference (univariate GLM with interaction term, *P* = 0.029). (**c**) *PR-1* expression in leaves of Col-0 plants growing on soil pre-conditioned by healthy or diseased Col-0 plants at 30 min, 4 h, and 6 h after dipping in 1 mM SA or tap water (control). *PR-1* expression is expressed as fold-change in SA-treated plants relative to mock-treated control plants growing on soil conditioned by healthy plants. Asterisk denotes a significant difference as determined by Student’s *t*-test (*P* < 0.05; n = 3).

Arabidopsis plants growing on natural Reijerscamp soil that has been pre-conditioned by a population of *Hpa*-infected Arabidopsis plants are more resistant to *Hpa* infection than plants growing on soil pre-conditioned by healthy plants (Fig. 1, Fig. S1)^12^. We hypothesized that the perception of such a SBL is the result of systemic resistance induced by microbes that are recruited by the infected plants. To test this, we first pre-conditioned Reijerscamp soil by growing Col-0 plants that were either infected by *Hpa* or not. Subsequently, we cultivated Col-0 and specific mutants impaired in known defense signaling pathways in the pre-conditioned soil and examined their level of resistance against *Hpa* infection*.* In the experimental setup, we used the *myb72* mutant that is blocked in its capacity to develop SA-independent ISR in response to various beneficial rhizobacteria and fungi^16^. Additionally, we used the SA-response mutants *npr1* and *sid2* because several beneficial rhizobacteria and fungi have been shown to activate an SA-dependent ISR response also known as systemic acquired resistance (SAR)^16,20^. Both Col-0 and *myb72* plants were significantly more resistant to *Hpa* infection when growing on soil that was pre-conditioned by *Hpa-*infected Col-0 plants than when growing on soil pre-conditioned by healthy Col-0 plants (Fig. 6a). However, neither *npr1* nor *sid2* mutant plants developed resistance against *Hpa* when grown on soil pre-conditioned by *Hpa*-infected Col-0 plants (Fig. 6b). This suggests that perception of the *Hpa-*mediated SBL involves systemic defense signaling in the response population of plants that depends on SA, but does not require MYB72.

To further corroborate this finding we monitored the expression of the SA-responsive marker gene *PR-1* before and after *Hpa* infection in Col-0 plants growing in SBL-conditioned and non-conditioned Reijerscamp soil. Col-0 plants transcribed significantly more of the SA-responsive marker gene *PR-1* in response to SA treatment when growing on soil pre-conditioned by diseased plants than on soil pre-conditioned by healthy plants (Fig. 6c). This suggests that defense responses of plants perceiving an *Hpa-*induced SBL are primed for SA-dependent defenses.

## Discussion

Recent studies show that plants can create a SBL in response to both biotic and abiotic stresses, resulting in the modulation of microbial communities in the rhizosphere that can act favorably on plant growth and immunity^12,13,53,54^. Here, we investigated biological mechanisms involved in the creation and perception of the SBL created by downy mildew-infected Arabidopsis plants.

### Creation of the SBL requires the coumarin biosynthesis genes *MYB72* and *F6’H1*

In our study, conditioning of a natural soil with *Hpa*-infected plants resulted in a SBL that consistently reduced disease severity in a subsequent *Hpa*-inoculated population of plants growing on this SBL-conditioned soil, compared to soil conditioned with healthy plants. We show that the coumarin biosynthesis genes *MYB72* and *F6’H1* are required for the creation of the *Hpa*-mediated SBL (Fig. 1) and identified MYB72- and F6’H1-dependent shifts in the rhizosphere microbial communities of *Hpa-*infected plants (Figs. 2–4) that may be responsible for the enhanced resistance of plants growing in SBL-conditioned soil. *Hpa* infection on the leaves of Col-0 plants resulted in dramatic metabolomic changes in the root exudates (Fig. 5), with increased levels of metabolite features representing a diversity of secondary and primary metabolites. Surprisingly, the MYB72- and F6’H1-dependent coumarins scopoletin, fraxetin and esculetin were not among them. For the main coumarin of Arabidopsis, scopeletin, we showed that its levels even decreased in root exudates of *Hpa-*infected plants. We, thus, hypothesize that either (1) a drop in coumarin secretion is responsible for the creation of the *Hpa-*induced SBL, allowing coumarin-sensitive microbes to increase in abundance, or (2) the tested coumarins scopoletin, fraxetin and esculetin are themselves not responsible for the creation of the *Hpa-*induced SBL, but other so-far unidentified MYB72- and F6’H1-dependent compounds play a role. An argument against hypothesis 1 is that *myb72* and *f6’h1* plants do not create a SBL that reduces *Hpa* disease severity in a response population of Col-0 plants to the extent that *Hpa-*infected Col-0 plants do (Fig. 1). Future, more detailed metabolite profiling of *Hpa-*infected Col-0, *myb72*, and *f6’h1-*plants should shed light on this matter.

### Foliar *Hpa* infection confers a shift in bacterial microbiota in the rhizosphere

Analysis of the root microbiome showed that the rhizosphere bacterial community is significantly affected by foliar *Hpa* infection. Moreover, the majority of bacteria that became more abundant on roots of Col-0 plants in response to *Hpa* infection were not promoted on the roots of *myb72* and *f6’h1* mutant plants, again underscoring that MYB72 and F6’H1 are essential for the creation of the *Hpa-*induced SBL. While the root bacterial community on Col-0 roots was significantly affected by foliar *Hpa* infection (Table S2), the root fungal community was not (Table S3). This suggests that bacteria and not fungi are responsible for the *Hpa*-triggered SBL. We identified a taxonomically diverse group of bacterial ASVs of which the abundance on the root was significantly affected by foliar *Hpa* infection in two independent experiments. The *Xanthomonas*, *Stenotrophomonas* and *Microbacterium* spp. rhizobacterial strains that were identified as being responsive to foliar *Hpa* infection in our previous study^12^ were not among them. Also in the present study, the overlap between the bacterial communities that significantly responded to foliar *Hpa* infection was not large (13 of the 162 MYB72- and/or F6’H1-dependent ASVs in Experiment 1 and 110 ASVs in Experiment 2 overlapped between the two experiments (Fig. S5). This suggests that the *Hpa-*induced SBL is not mediated by a select set of defined microbial species, but merely represents a shift in taxa representing microbes that either alone or in concerted action with other microbes in the microbial community are able to confer enhanced resistance against *Hpa* infection in a subsequent population of plants. In our previous study (Berendsen et al.^18^), the identified and selected *Xanthomonas, Stenotrophomonas* and *Microbacterium* spp. strains were demonstrated as a consortium to enhance biofilm formation and to consistently confer enhanced resistance against *Hpa* infection. This suggests that this small consortium of microbes can be responsible for a *Hpa-*induced SBL that effectively enhances resistance in a next generation of plants. However, the present study shows that depending on the microbial context in the soil, other microbial taxa may as well be capable of creating an effective SBL.

One of the candidate microbial taxa that consistently emerged in this study as being enriched on roots of *Hpa-*infected Col-0 plants and not on coumarin-deficient mutant plants represents a *Massilia* sp. (*Massilia* ASV 09023; Figs. 3, 4 and Fig. S5). *Massilia* is a genus of bacteria that is abundantly found in soils. Moreover, increased abundance of *Massilia* has previously been associated with the induction of SA signaling-dependent resistance of rice plants against bacterial pathogen *Xanthomonas oryzae*^55^. Future research will be focused on identifying the *Massilia* sp. strain(s) involved in the creation of the SBL and investigate the potential for their use as a bioinoculant in agriculture.

### Perception of the SBL induces a SA-dependent disease resistance

Because the *Hpa-*induced SBL leads to enhanced protection against a foliar pathogen, it is likely that this is conferred via rhizobacteria-mediated ISR. Activation of ISR by the well-characterized beneficial rhizosphere bacterium *P. simiae* WCS417 and several other beneficial root-associated microbes requires the transcription factor MYB72 and functions independently of SA^16,21^. Other beneficial microbes, however, have been found to systemically induce disease resistance that is SA-dependent^56–58^. Our results show that the *Hpa-*mediated SBL induces a systemic resistance in the responding population of plants that does not require MYB72, but rather depends on SA-dependent signaling and involves priming of SA-responsive defenses. Thus, collectively, our results show that downy mildew infection of a first population of plants consistently results in a MYB72/F6’H1-dependent shift in the bacterial rhizosphere microbiome that is capable of inducing SA-dependent defense priming in a subsequent response population of plants, leading to increased resistance to the pathogen that initiated the SBL. From an ecological and agricultural perspective, this would mean that a diseased plant can provide protection to a next generation of plants growing in the conditioned soil by creating a microbial SBL that induces systemic immunity against the attacking pathogen. Interestingly, it has been demonstrated that soil, that was taken from fields in Washington state that were suppressive to either take-all or Rhizoctonia root rot of wheat, induced resistance in Arabidopsis plants to foliar *Pseudomonas syringae* infection. This shows that, also in the field, soils conditioned by leaf-infected plants can trigger ISR in a subsequent generation of plants^59^. Elucidating the mechanistic basis of pathogen-induced SBLs for both the involvement of the plant and the recruited microbiome will be highly instrumental for the development of more sustainable microbiome-assisted agricultural practices.

## Supplementary Information

Supplementary Information.

Supplementary Data S1.

Supplementary Data S2.

Supplementary Data S3.

## Data Availability

The data that support the findings of this study are available from the corresponding author upon reasonable request. Moreover, the raw sequence data generated of this study are available at https://www.ncbi.nlm.nih.gov/bioproject/838813.
