# Supplementary material for: Coumarin biosynthesis genes are required after foliar pathogen infection for the creation of a microbial soil-borne legacy that primes plants for SA-dependent defenses
Source: Sci Rep. 2022 Dec 28;12:22473. doi: 10.1038/s41598-022-26551-x (PMC9797477; doi:10.1038/s41598-022-26551-x)
Supplement: Supplementary file 1 — Supplementary Information. [file 41598_2022_26551_MOESM1_ESM.docx]

## Supporting Information

Article title: **Coumarin biosynthesis genes are required after foliar pathogen infection for the creation of a microbial soil-borne legacy that primes plants for SA-dependent defenses**

Authors: Gilles Vismans, Sietske van Bentum, Jelle Spooren, Yang Song, Pim Goossens, Josep Valls, Basten L. Snoek, Benjamin Thiombiano, Mario Schilder, Lemeng Dong, Harro J. Bouwmeester, Pierre Petriacq, Corné M.J Pieterse, Peter A.H.M. Bakker and Roeland L. Berendsen

**The following Supporting Information is available for this article:**

**Fig. S1** Coumarin biosynthesis is essential for the creation of an Hpa-induced SBL that enhances resistance against downy mildew in a responding population of plants.

**Fig. S2** Aboveground *Hpa* infection does not affect α-diversity of root bacterial communities.

**Fig. S3** Aboveground Hpa infection does not affect the fungal root community.

**Fig. S4** Effect of aboveground Hpa infection on root bacterial communities in Experiment 2.

**Fig. S5** ASVs responding to Hpa infection in Experiment 1 and 2.

**Table S1** Phasing primers adapted from De Muinck et al (2017).

**Table S2** Pairwise statistical comparison by PERMANOVA of root bacterial communities of Experiment 1.

**Table S3** Pairwise statistical comparison by PERMANOVA of root fungal communities of Experiment 1.

**Table S4** Pairwise statistical comparison by PERMANOVA of root bacterial communities of Experiment 2.

**Methods S1**

**Data S1** (Separate file)

**Data S2** (Separate file)

**Data S3** (Separate file)


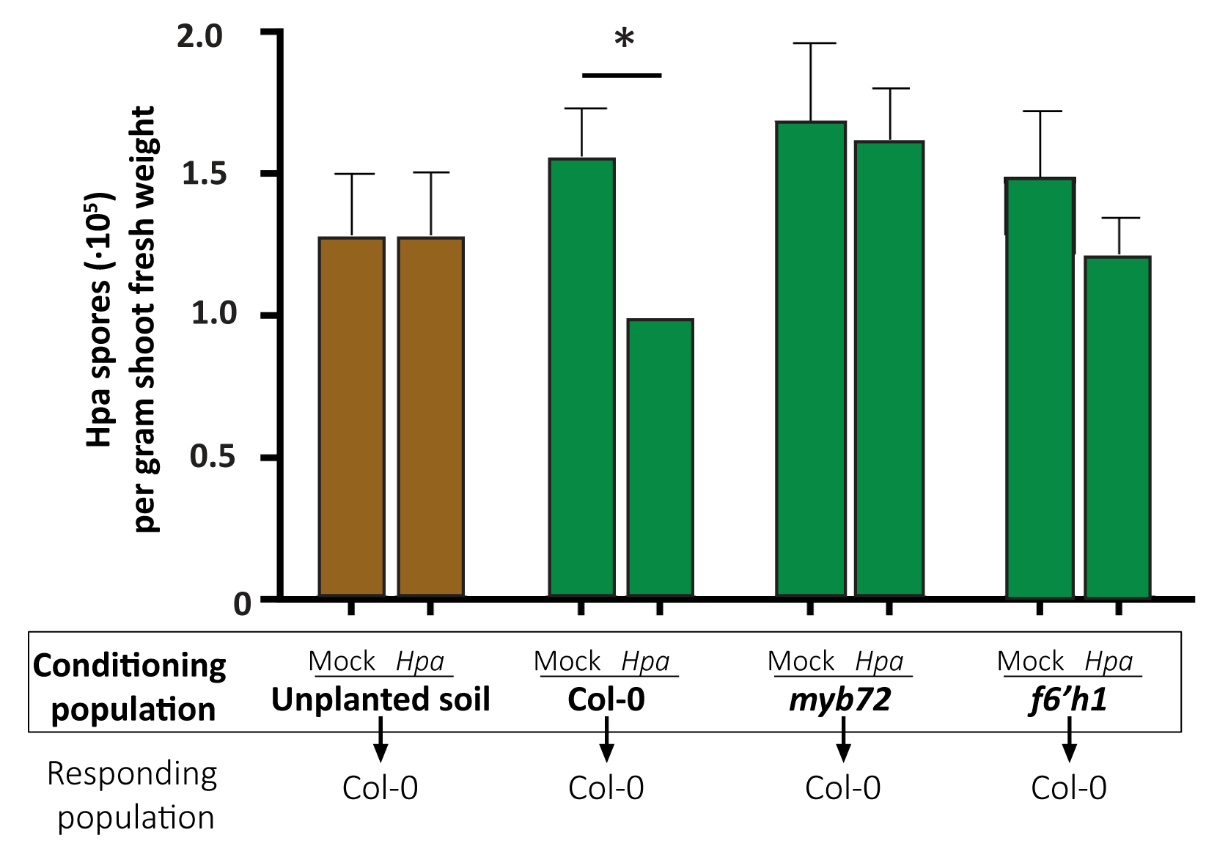


**Fig. S1** **Coumarin biosynthesis is essential for the creation of a *Hpa-*induced SBL that enhances resistance against downy mildew in a responding population of plants.** Results are an independent replication of the experiment shown in Fig 1**.** Green bar graphs represent the disease severity of SBL-responding populations of Col-0 plants growing on natural Reijerscamp soil pre-conditioned with mock-treated or *Hpa-*inoculated Col-0, *myb72*, or *f6’h1* seedlings). Brown bar graphs represent the disease severity of responding populations of Col-0 plants growing in control pots that remained unplanted during the conditioning phase and were mock- or *Hpa*-inoculated directly on the soil. Bars depict the average *Hpa* spore production per gram of shoot fresh weight (n = 10). Error bars depict standard error of the mean. Asterisk denotes a significant difference (*P* = 0.01 in Students *t*-test).

**Fig. S2 Aboveground *Hpa* infection does not affect α-diversity of root bacterial communities.**

Boxplot of the Shannon diversity index of the bacterial communities in unplanted soil and the rhizospheres of three-week-old Col-0 , *f6’h1* and *myb72* plants. Samples were taken one week after either mock treatment or inoculation with *Hpa*.


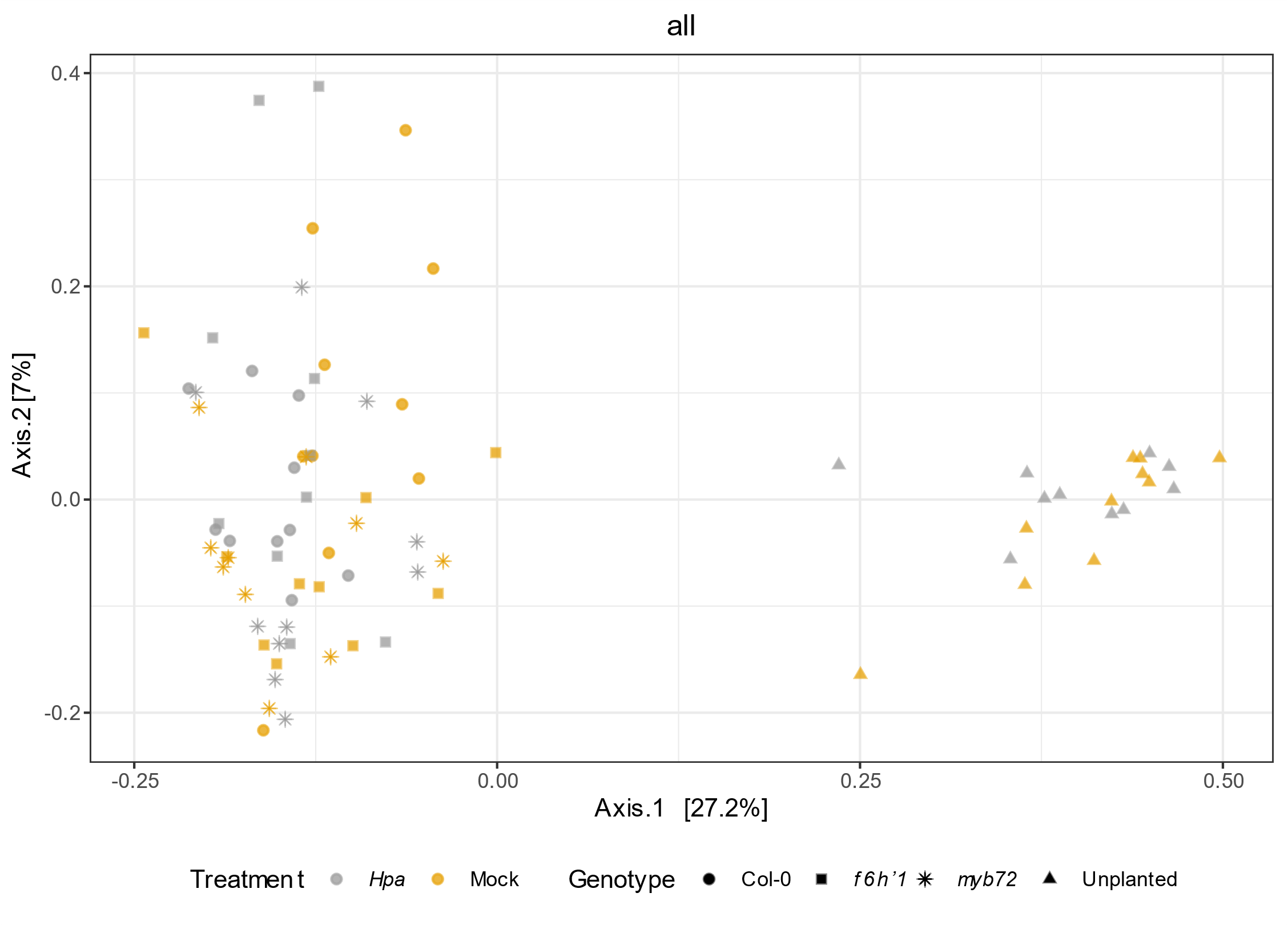


**Fig. S3** **Aboveground *Hpa* infection does not affect the fungal root communities.** Principal coordinate analysis (PCoA) of fungal communities in unplanted soil (▲), or the rhizospheres of three-week-old Col-0 (●), *myb72* (⁎) and *f6’h1* (■) plants. Samples were taken 1 week after either mock treatment (yellow symbols) or inoculation with *Hpa* (grey symbols).


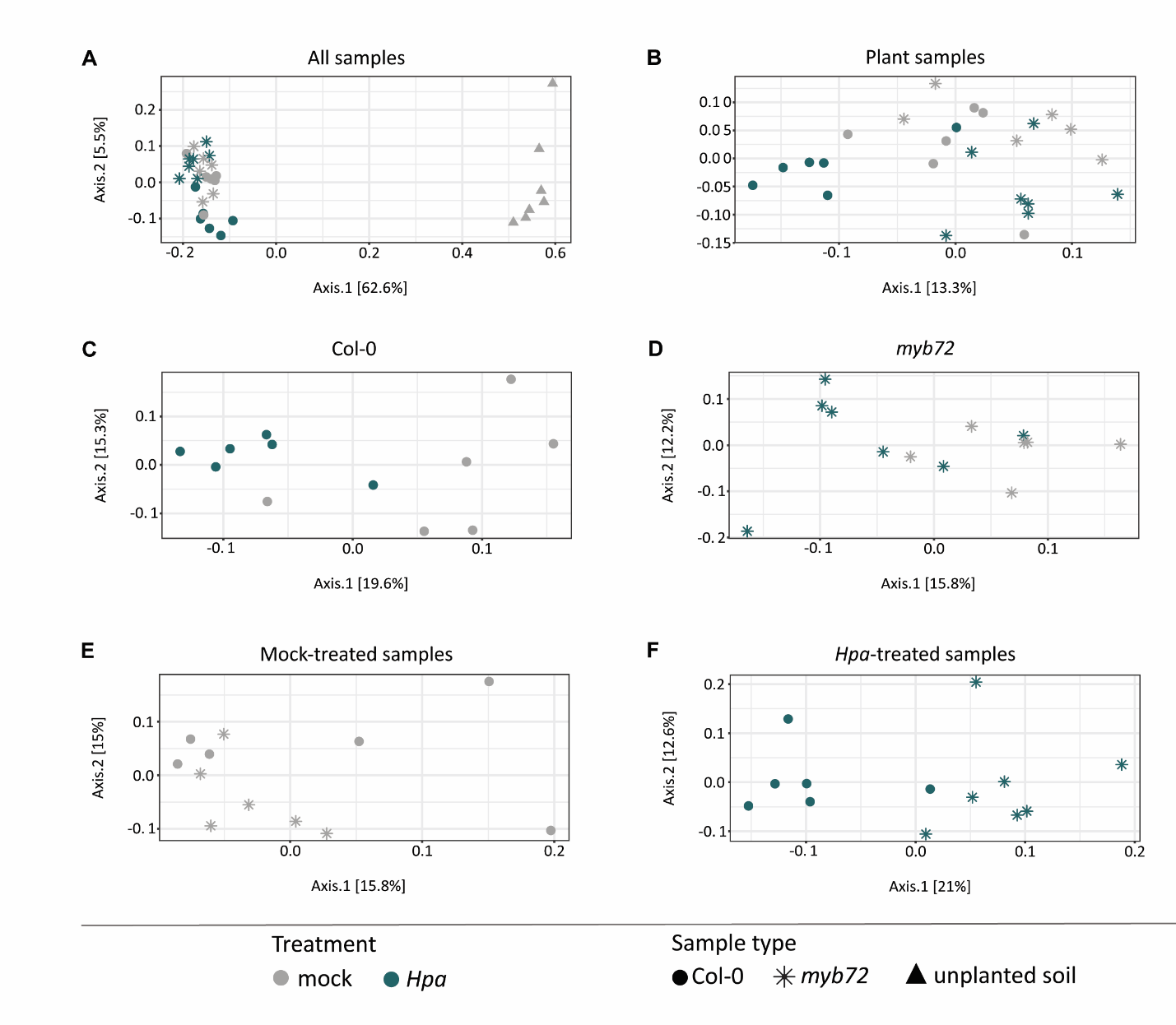


**Fig. S4** **Effect of aboveground Hpa infection on root bacterial communities in Experiment 2. A)** Principal coordinate analysis (PCoA) of bacterial communities in unplanted soil (▲) and the rhizospheres of three-week-old Col-0 (●), *myb72* (⁎) plants. Samples were taken one week after either mock treatment (grey symbols) or inoculation with *Hpa* (blue-green symbols). **B)** PCoA of only the rhizosphere samples. C) PCoA of only the Col-0 samples. **D)** PCoA of only the *myb72* samples. **E)** PCoA of root bacterial communities of mock-treated Col-0 (●)and *myb72* (⁎) plants. **F)** PCoA of root bacterial communities of *Hpa*-infected Col-0 (●) and *myb72* (⁎) plants.

**
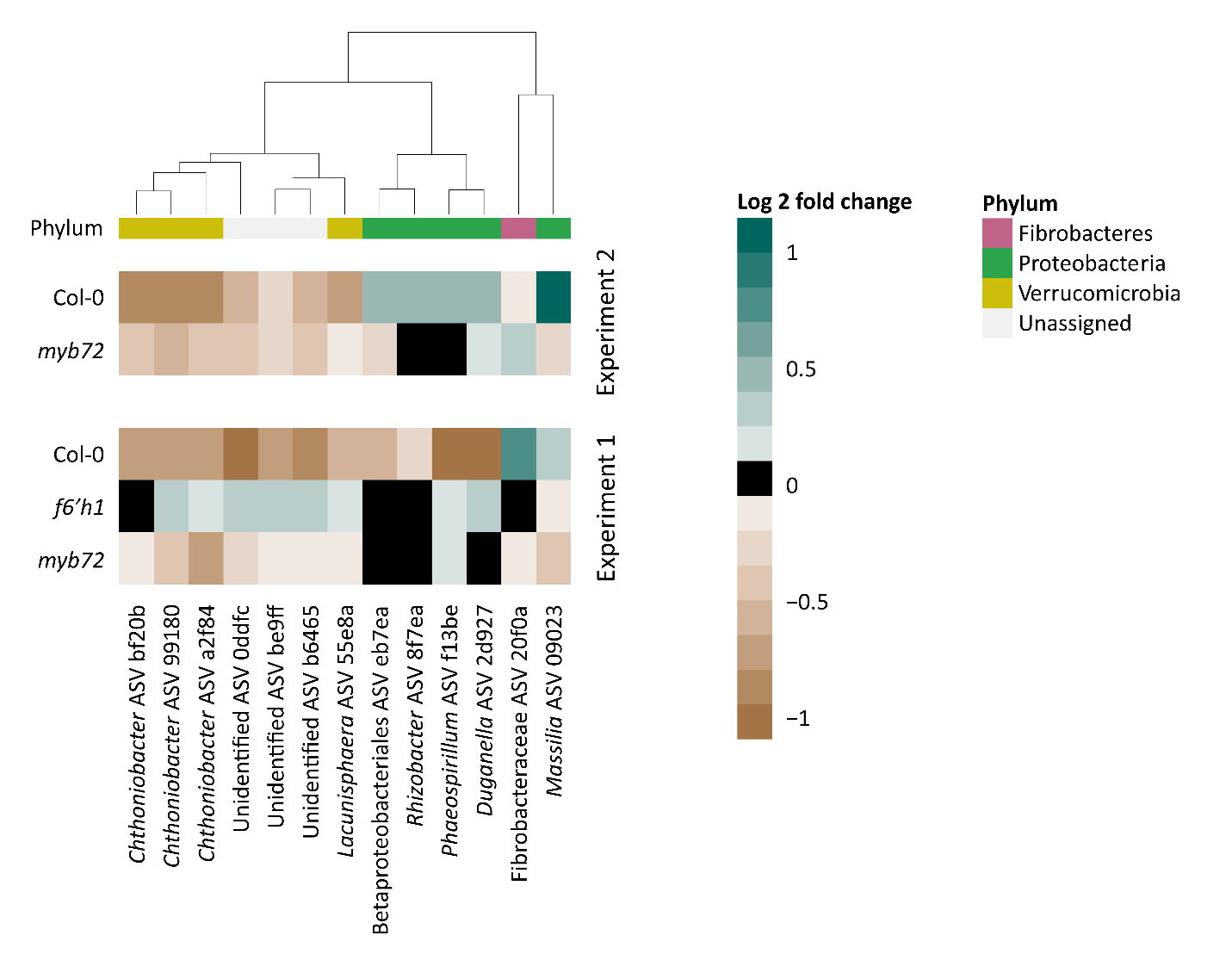
**

**Fig. S5** **ASVs responding to *Hpa*infection in Experiment 1 and 2.**Fold change of 13 ASVs on Col-0,  *myb72* and *f6’h1* plants. The 13 ASVs are differentially abundant between mock- and *Hpa*-treated Col-0 plants in both Experiment 1 and 2. Shrunk log-2-fold change in abundance between mock- and *Hpa*-treated plants is shown as calculated with DESeq2. Colors above the heatmap indicate phylum annotation. Only *Massilia* ASV 09023 increases in abundance on wild type roots following foliar *Hpa* inoculation in both experiments, but decreases in response to host infection on mutant plants.

| **Table S1.** Phasing primers adapted from De Muinck *et al* (2017)*.* | |
| --- | --- |
| **Name** | **Sequence (5' - 3')** |
| NGS1-16s-N701 | TCGTCGGCAGCGTCAGATGTGTATAAGAGACAGTCGCCTTACCTGTGGCCTACGGGNGGCWGCAG |
| NGS1-16s-N702 | TCGTCGGCAGCGTCAGATGTGTATAAGAGACAGCTAGTACGGAGTGGCCTACGGGNGGCWGCAG |
| NGS1-16s-N703 | TCGTCGGCAGCGTCAGATGTGTATAAGAGACAGTTCTGCCTTGCGACCTACGGGNGGCWGCAG |
| NGS1-16s-N704 | TCGTCGGCAGCGTCAGATGTGTATAAGAGACAGGCTCAGGAATGACCTACGGGNGGCWGCAG |
| NGS1-16s-N705 | TCGTCGGCAGCGTCAGATGTGTATAAGAGACAGAGGAGTCCCGACCTACGGGNGGCWGCAG |
| NGS1-16s-N706 | TCGTCGGCAGCGTCAGATGTGTATAAGAGACAGCATGCCTACGACCTACGGGNGGCWGCAG |
| NGS1-16s-N707 | TCGTCGGCAGCGTCAGATGTGTATAAGAGACAGGTAGAGAGGTCCTACGGGNGGCWGCAG |
| NGS1-16s-N708 | TCGTCGGCAGCGTCAGATGTGTATAAGAGACAGCCTCTCTGGTCCTACGGGNGGCWGCAG |
| NGS1-16s-N709 | TCGTCGGCAGCGTCAGATGTGTATAAGAGACAGAGCGTAGCTCCTACGGGNGGCWGCAG |
| NGS1-16s-N710 | TCGTCGGCAGCGTCAGATGTGTATAAGAGACAGCAGCCTCGTCCTACGGGNGGCWGCAG |
| NGS1-16s-N711 | TCGTCGGCAGCGTCAGATGTGTATAAGAGACAGTGCCTCTTCCTACGGGNGGCWGCAG |
| NGS1-16s-N712 | TCGTCGGCAGCGTCAGATGTGTATAAGAGACAGTCCTCTACCCTACGGGNGGCWGCAG |
| NGS1-16s-N501 | GTCTCGTGGGCTCGGAGATGTGTATAAGAGACAGTAGATCGCCACTTCTGACTACHVGGGTATCTAATCC |
| NGS1-16s-N502 | GTCTCGTGGGCTCGGAGATGTGTATAAGAGACAGCTCTCTATTTCTCTGACTACHVGGGTATCTAATCC |
| NGS1-16s-N503 | GTCTCGTGGGCTCGGAGATGTGTATAAGAGACAGTATCCTCTACTCAGACTACHVGGGTATCTAATCC |
| NGS1-16s-N504 | GTCTCGTGGGCTCGGAGATGTGTATAAGAGACAGAGAGTAGAGATAGACTACHVGGGTATCTAATCC |
| NGS1-16s-N505 | GTCTCGTGGGCTCGGAGATGTGTATAAGAGACAGGTAAGGAGCTAGACTACHVGGGTATCTAATCC |
| NGS1-16s-N506 | GTCTCGTGGGCTCGGAGATGTGTATAAGAGACAGACTGCATATCGACTACHVGGGTATCTAATCC |
| NGS1-16s-N507 | GTCTCGTGGGCTCGGAGATGTGTATAAGAGACAGAAGGAGTAAGACTACHVGGGTATCTAATCC |
| NGS1-16s-N508 | GTCTCGTGGGCTCGGAGATGTGTATAAGAGACAGCTAAGCCTGACTACHVGGGTATCTAATCC |

**Table S2.** Pairwise statistical comparison by PERMANOVA of root bacterial communities of Experiment 1. Bacterial communities are compared per plant genotype (Col-0, *myb72, f6’h1*) and treatment (mock vs *Hpa*-inoculated) and data corresponds to Fig. 1-3. The R^2^ and *P*-values for each comparison are shown in the table.

|  | Col-0 Mock | Col-0  *Hpa* | *myb72*  Mock | *myb72*  *Hpa* | *f6’h1*  Mock | *f6’h1*  *Hpa* | Unplanted  Mock | Unplanted  *Hpa* |
| --- | --- | --- | --- | --- | --- | --- | --- | --- |
| Col-0 Mock |  | *P* = 0.009 | *P* = 0.009 | *P* = 0.019 | *P* = 0.004 | *P* = 0.008 | *P* = 0.002 | *P* = 0.002 |
| Col-0  *Hpa* | R^2^ = 0.14 |  | *P* = 0.012 | *P* = 0.081 | *P* = 0.065 | *P* = 0.016 | *P* = 0.002 | *P* = 0.002 |
| *myb72*  Mock | R^2^ = 0.14 | R^2^ = 0.12 |  | *P* = 0.020 | *P* = 0.149 | *P* = 0.050 | *P* = 0.002 | *P* = 0.002 |
| *myb72*  *Hpa* | R^2^ = 0.13 | R^2^ = 0.12 | R^2^ = 0.11 |  | *P* = 0.047 | *P* = 0.320 | *P* = 0.002 | *P* = 0.002 |
| *f6’h1*  Mock | R^2^ = 0.17 | R^2^ = 0.10 | R^2^ = 0.07 | R^2^ = 0.10 |  | *P* = 0.020 | *P* = 0.002 | *P* = 0.002 |
| *f6’h1*  *Hpa* | R^2^ = 0.13 | R^2^ = 0.12 | R^2^ = 0.09 | R^2^ = 0.08 | R^2^ = 0.10 |  | *P* = 0.002 | *P* = 0.002 |
| Unplanted  Mock | R^2^ = 0.71 | R^2^ = 0.72 | R^2^ = 0.74 | R^2^ = 0.72 | R^2^ = 0.73 | R^2^ = 0.75 |  | *P* = 0.319 |
| Unplanted  *Hpa* | R^2^ = 0.75 | R^2^ = 0.76 | R^2^ = 0.77 | R^2^ = 0.76 | R^2^ = 0.77 | R^2^ = 0.78 | R^2^ = 0.06 |  |

**Table S3.** Pairwise statistical comparison by PERMANOVA of root fungal communities of Experiment 1. Fungal communities are compared per plant genotype (Col-0, *myb72*, *f6’h1*) and treatment (mock vs *Hpa*-inoculated). Data corresponds to the PCoA in Fig. S2. The R^2^ and *P*-values for each comparison are shown in the table.

|  | Col-0 Mock | Col-0  *Hpa* | *myb72*  Mock | *myb72*  *Hpa* | *f6’h1*  Mock | *f6’h1*  *Hpa* | Unplanted  Mock | Unplanted  *Hpa* |
| --- | --- | --- | --- | --- | --- | --- | --- | --- |
| Col-0  Mock |  | *P* = 0.23 | *P* = 0.23 | *P* = 0.47 | *P* = 0.28 | *P* = 0.45 | *P* = 0.02 | *P* = 0.02 |
| Col-0  *Hpa* | R^2^ = 0.08 |  | *P* = 0.47 | *P* = 0.28 | *P* = 0.25 | *P* = 0.47 | *P* = 0.02 | *P* = 0.02 |
| *myb72*  Mock | R^2^ = 0.07 | R^2^ = 0.05 |  | *P* = 0.71 | *P* = 0.71 | *P* = 0.28 | *P* = 0.02 | *P* = 0.02 |
| *myb72*  *Hpa* | R^2^ = 0.06 | R^2^ = 0.07 | R^2^ = 0.05 |  | *P* = 0.71 | *P* = 0.38 | *P* = 0.02 | *P* = 0.02 |
| *f6’h1*  Mock | R^2^ = 0.07 | R^2^ = 0.07 | R^2^ = 0.05 | R^2^ = 0.05 |  | *P* = 0.24 | *P* = 0.02 | *P* = 0.02 |
| *f6’h1*  *Hpa* | R^2^ = 0.06 | R^2^ = 0.06 | R^2^ = 0.07 | R^2^ = 0.06 | R^2^ = 0.08 |  | *P* = 0.02 | *P* = 0.02 |
| Unplanted  Mock | R^2^ = 0.34 | R^2^ = 0.39 | R^2^ = 0.40 | R^2^ = 0.34 | R^2^ = 0.36 | R^2^ = 0.37 |  | *P* = 0.34 |
| Unplanted  *Hpa* | R^2^ = 0.32 | R^2^ = 0.38 | R^2^ = 0.38 | R^2^ = 0.33 | R^2^ = 0.35 | R^2^ = 0.36 | R^2^ = 0.06 |  |

|  |
| --- |
| \|  \| Col-0  Mock \| Col-0  *Hpa* \| *myb72*  Mock \| *myb72*  *Hpa* \| Unplanted  Mock \| \| --- \| --- \| --- \| --- \| --- \| --- \| \| Col-0 Mock \|  \| *P* = 0.013 \| *P* = 0.098 \| *P* = 0.037 \| *P* = 0.003 \| \| Col-0 *Hpa* \| R^2^ = 0.15 \|  \| *P* = 0.013 \| *P* = 0.004 \| *P* = 0.003 \| \| *myb72* Mock \| R^2^ = 0.11 \| R^2^ = 0.21 \|  \| *P* = 0.013 \| *P* = 0.003 \| \| *myb72 Hpa* \| R^2^ = 0.11 \| R^2^ = 0.17 \| R^2^ = 0.12 \|  \| *P* = 0.004 \| \| Unplanted Mock \| R^2^ = 0.72 \| R^2^ = 0.76 \| R^2^ = 0.74 \| R^2^ = 0.73 \|  \|   **Table S4.** Pairwise statistical comparison by PERMANOVA of root bacterial communities of Experiment 2. Bacterial communities are compared per combination of plant genotype (Col-0, *myb72*) and treatment (mock vs *Hpa*-inoculated) and data corresponds to the PCoAs in Fig. S3. The R^2^ and *P*-values for each comparison are shown in the table |
|  |

**Methods S1**

# **SBL experiments**

In this study, natural soil collected from the Reijerscamp nature reserve in the Netherlands was used as previously described (Berendsen *et al.*, 2018). The SBL setup was performed as previously described in detail (Vismans *et al.*, 2021). Briefly, 60-ml pots were filled with approximately 120 g of Reijerscamp soil. To prevent algae and moss growth, a non-transparent, perforated plastic cover was placed on the soil. The covers were made by cutting a circular shape from a micro pipette tip holder (Greiner Bio-One, 0,5-10 µl, Item No.: 771280). Arabidopsis seeds were imbibed by suspending seeds in 0.2% (w/v) water agar in the dark at 4°C for 2 days. Using a 1000-µl pipette, 1-2 seeds were pipetted in each hole of the cover, resulting in approximately 30 seeds per pot. Each pot was placed on an individual saucer and randomly placed in trays covered by transparent lids. The trays were placed in a climate-controlled plant growth chamber at 21°C, 70% relative humidity, 10-h light/14-h dark cycles, and at a light intensity of 100 µmol m^-2^ s^-1^. After one week, the lids were changed for lids with a mesh to reduce humidity. Two weeks after sowing, half of the pots were inoculated with a 50 spores/µl suspension of *Hyaloperonospora* *arabidopsidis* noco2 (*Hpa)* as previously described (Asai *et al.*, 2015). The other pots were sprayed with tap water as mock treatment. The mesh lids were replaced by the transparent lids to increase humidity. *Hpa* symptoms were allowed to develop for one week, after which all aboveground material of this so-called “conditioning population” of plants was cut off using a razor blade. A new population of plants was sown and grown as described above. After two weeks of growth of this second plant population, all plants were inoculated with *Hpa* and symptoms were allowed to develop for one week. Disease severity in this so-called SBL “responding population” of plants was determined based on *Hpa* spore densities normalized for plant fresh weight (details below).

Wild-type plants of the Arabidopsis Col-0 accession were used in both the conditioning phase of the experiment (1^st^ population of plants) and in the response phase (2^nd^ population of plants). Depending on the experiment, Col-0 wild-type plants were replaced by mutant *myb72-2,* which is impaired in both rhizobacteria-mediated ISR (Van der Ent *et al.*, 2008) and in the biosynthesis and secretion of coumarins (Zamioudis *et al.*, 2014; Stringlis *et al.*, 2018), the coumarin biosynthesis mutant *f6’h1,* which is impaired in the coumarin biosynthesis enzyme Feruloyl-CoA 6'-Hydroxylase1 (Schmid *et al.*, 2014)*,* or by SA signaling mutants *npr1-1* (Cao *et al.*, 1997) or *sid2-1* (Wildermuth *et al.*, 2001). As controls, pots were left unplanted in either the conditioning phase or the response phase of the experiment. All experiments were performed at least twice with similar results.

1. **Quantification of *Hpa* spore production**

To quantify *Hpa* infection, the number of spores produced per gram of shoot fresh weight was determined. To this end, aboveground plant parts were cut using a razor blade and placed in a 15-ml Greiner tube, containing 3 ml of water, after which shoot weight was measured. After shaking, three separate 1-µl drops were pipetted onto a microscope slide. Spores were counted in each of the individual droplets using a light microscope at 100x magnification.

1. **Defense priming assay**

To test for priming of plant defenses, 2-week-old plants in the response phase of an SBL experiment were dipped in a 1 mM SA solution supplemented with 0.015% Silwet L-77 or in a mock solution containing 0.015% Silwet L-77. Plants were harvested at 30 min, 4 h, and 6 h after dipping, immediately snap frozen in liquid nitrogen and stored at -80°C until further processing. RNA extraction was performed as described by Oñate-Sánchez (2008) with minor modifications. Quantitative real-time PCR (qPCR) analysis of the defense-related marker gene *PATHOGENESIS-RELATED1* (*PR1*) was performed as described by Van Wees *et al.* (2013).

1. **Soil-borne legacy microbiome analysis**

At the end of the SBL conditioning phase, after the shoots of the first population of plants were removed, the roots from at least ten replicate pots were collected for each treatment. Each pot was emptied on a 1-mm^2^ mesh sieve and roots were subsequently cleaned under running tap water. When most of the soil was washed away, the roots were collected with tweezers in Qiagen PowerBead tubes, snap frozen and stored at -80°C until further processing. DNA was extracted using the DNeasy® Powerlyzer® Powersoil® kit (product #: 12855-100). The protocol was followed according to the manufacturer’s instructions, with the adjustment of a 10-min incubation step at 60°C after the addition of solution C1. For the bead beating option in step 4, two times 10 min at 30 Hz in TissueLyser II (Qiagen) was used. DNA concentrations were determined with a Thermofisher Nanodrop® 2000 and set to 5 ng/µl prior to preparation of the amplicon sequencing library.

1. **Preparation of amplicon sequencing library**

The *16S rRNA* gene amplicon libraries were constructed using the 16S metagenomic sequencing library protocol for the Illumina MiSeq system (Illumina, 2013) using 16S V3-V4 phasing primers adpted from (De Muinck *et al.* (2017; Table S4). Moreover, the barcoded PCR primers described by Baym and co-workers (2015) were used in a total PCR reaction volume of 25 µl instead of 50 µl. Three µl of the purified samples was run on a 1% agarose gel to check the amplicon size and the concentration of double-stranded DNA was determined with the Qubit Broad-Range kit (Thermofisher Scientific). Each sample was adjusted to 2 ng/µl and pooled in a 1:1 ratio. The pooled library was sent for sequencing on a 2x300 MiSeq at the USEQ sequencing facility (Utrecht University, the Netherlands). The fungal amplicon libraries were constructed in the same way but using primers targeting the internal transcribed spacer (ITS) region 2 of the ribosome encoding genes (Ihrmark *et al.*, 2012).

1. **Amplicon sequence data pre-processing**

The 16S amplicon sequence data was analyzed using Qiime2 version 2019.7 (Bolyen *et al.*, 2019). Primers were removed using the Cutadapt (Martin, 2011) plugin of Qiime2 with standard settings. For Experiment 1 , forward and reverse reads were truncated at 260 bp and 215 bp, respectively. For Experiment 2 (with genotypes Col-0 and *myb72*), forward and reverse reads were truncated at 272 bp and 215 bp, respectively. Denoising, dereplicating, chimera removal and paired-end joining was performed with DADA2 resulting in a final alignment at 100% similarity, giving amplicon sequence variants (ASVs) (Callahan *et al.*, 2016). A VSEARCH-based consensus taxonomy classifier was used (Rognes *et al.*, 2016) with the SILVA database version 128 (Quast *et al.*, 2013; Yilmaz *et al.*, 2014) for bacterial taxonomy assignment. For Experiment 1, six samples with a sequencing bias, all produced with index primer N705, were removed from the dataset, resulting in a total of 67 samples, of which 19 were bulk soil samples. Experiment 2 comprised 32 samples after pre-processing, of which 7 were bulk soil samples. ASVs were filtered with total relative abundance ≥ 0.01% and detection in at least 10% of the samples. Furthermore, reads annotated as chloroplast or mitochondria were removed.

For the ITS amplicon sequence data a similar approach was taken as for the 16S data, with some adaptations. As DADA2 removes untruncated reads and this could result in the removal of short, but high quality ITS reads being removed, the reverse compliment of the ITS primers was removed when present to avoid the removal of such short ITS reads. The UNITE reference database (v.8.0) was used to assign taxonomy to the reads. After removing low read samples and filtering for ASVs with a total relative abundance of abundance ≥ 0.01% and detection in at least 10% of samples, there were 74 samples left.

1. **Differential abundance analysis**

Five statistical methods were used for differential abundance testing of ASVs: ANCOM-bc, DESeq2, Fisher’s exact test, Simper analysis and Spearman rank correlations (Clarke, 1993; Love et al., 2014; Lin and Peddada, 2020). ASVs were considered differentially abundant if the FDR-adjusted *P* ≤ 0.05 as generated by ANCOM-bc, DESeq2, and Spearman rank correlations. For Fisher’s exact test and Simper analysis we used unadjusted *P*≤ 0.05. Sparse ASVs denoted as structural zeroes by ANCOM-bc were ignored in downstream analysis unless also detected by at least one other statistical method.

1. **Software used**

All statistical analyses on the 16S amplicon sequencing data were performed in RStudio version 3.5.0 (data in Fig. 1 and 6) and version 3.6.1 (Fig. 2-4). Figures were created in RStudio version 3.6.1 using phyloseq (McMurdie and Holmes, 2013), ggplot2 (Wickham, 2016), tidyheatmap (<https://github.com/jbengler/tidyheatmap>). PRISM8 Graphpad and Adobe Illustrator CC 2017 and 2022 were used to prepare graphs and figures.

1. **Untargeted metabolomics**

Sterile root exudates secreted by Arabidopsis roots in response to foliar *Hpa* inoculation were collected as described by Song *et al*. (2021). Briefly, plants were sown on seed holders filled with agar-solidified 1/10 strength Hoagland (HG) solution (0.5 mM KNO_3_, 0.2 mM MgSO_4_, 0.2 mM Ca(NO_3_)_2_, 0.25 mM KH_2_PO_4_, 7 μM H_3_BO_3_, 1.4 μM MnCl_2_, 0.1 μM ZnSO_4_, 0.05 μM CuSO_4_, 1 μM, NaCl, 0.02 μM Na_2_MoO_4_, 5 μM Fe-EDTA, 0.47 mM MES, 1% agar, pH 5.8). Roots of the germinating seedlings penetrated the agar to grow into vials with full strength HG medium (5 mM KNO_3_, 2 mM MgSO_4_, 2 mM Ca(NO_3_)_2_, 2.5 mM KH_2_PO_4_, 70 μM H_3_BO_3_, 14 μM MnCl_2_, 1 μM ZnSO_4_, 0.5 μM CuSO_4_, 10 μM, NaCl, 0.2 μM Na_2_MoO_4_, 50 μM Fe-EDTA, 4.7 mM MES, pH 5.8) and were grown in controlled-climate conditions (21°C, 70% relative humidity, 16 h light/8 h dark, 100 µmol m^-2^ s^-1^ light intensity). The plants were kept sterile in Eco2boxes (Eco2 NV). After 19 days, leaves of half of the plants were carefully inoculated with 1-µl droplets of an *Hpa* spore suspension. Five days post-inoculation, plants were removed from the HG medium. A subsample of the medium was plated on agar-solidified Tryptic Soy Broth to check whether the root exudates were sterile, whereas the rest of the medium was immediately frozen for metabolomic analysis. Contaminated root exudates were discarded and sterile root exudates were lyophilized. Dried exudates (10 mg dried weight) were resuspended in 100 µl of methanol:water:formic acid (50:49.9:0.1, v/v), sonicated at 4°C for 20 min, vortexed and centrifuged (15 min, 14000 *g*, 4°C) to remove potential particles that could impede chromatographic separation. The final supernatants (80 µl) were transferred into glass vials containing a glass insert before injection through the liquid chromatography-mass spectrometry (LCMS) system (Pétriacq et al., 2017). Quality control samples were also prepared by mixing 5 µl of each sample into one single vial. This controls the analytical reliability of the LCMS system and allows filtering of the less robust metabolomic features (Luna *et al.*, 2020).

Untargeted metabolomics was conducted on an ACQUITY ultra high performance liquid chromatography (UHPLC) coupled to a SYNAPT G2Si quadrupole time-of-flight (Q-TOF) mass spectrometer equipped with an electrospray (ESI) source (Waters) as previously described (Pétriacq et al., 2017). Briefly, reverse-phase chromatographic separation was realized using a UPLC BEH C18 column (2.1 x 50 mm, 1.7 μm; Waters) with a pre-column of same chemistry phase at a flow rate of 400 μL min^−1^, and a mobile phase containing water with 0.05% formic acid (solution A), and acetonitrile with 0.05% formic acid (solution B) with the following gradient: 0-3 min 5-35% solution B, 3-6 min 35-100% solution B, holding at 100% solution B for 2 min, 8-10 min 100-5% solution B. The column temperature was set to 45°C and 10 μL of sample was injected into the system. Blank samples (methanol:water, 50:50, v:v) were also injected throughout the analytical batch.

MS detection by SYNAPT G2si was operated in negative ESI and sensitivity mode with a scan time of 0.2 s using the MS full scan (no collision energy). In addition, detected ions were ramped in the transfer cell in elevated energy mode (5 to 45 eV) following a Sequential Windowed Acquisition of all Theoretical Fragment ion (SWATH) MS method. SWATH generates Data Independent Acquisition (DIA) fragmentation patterns (MS^E^), thus allowing a thorough exploration of the MS data and facilitating the annotation of metabolomic features. The instrument operated over a mass range of 20 - 1200 Da with the following conditions: capillary voltage - 3 kV, sampling cone voltage - 60 V, source temperature 120°C, desolvation temperature 350°C, desolvation gas flow 800 L h^-1^, cone gas flow 60 L h^-1^. Prior to analyses, the instrument was calibrated with a sodium formate solution. In addition, infusion of leucine enkephalin as an internal reference (lockmass) ensured accurate mass measurements for each run. MassLynx v 4.1 (Waters) was used to control the system.

**Processing of untargeted metabolomic data**

Raw LCMS data were processed following the DIA MS2 deconvolution method using MS-DIAL software (v. 4.7; Tsugawa *et al.*, 2015). Optimized parameters were applied (MS1, tolerance, 0.01 Da; MS2 tolerance, 0.05 Da; retention time begin, 0 min; retention time end, 10 min; minimum peak height, 3000; mass slice width, 0.1 Da; smoothing (Savitzky–Golay) level, 4 scans; minimum peak width, 5 scans; sigma window value, 0.5). This enabled the detection of 923 Retention Time (RT)-*m/z* pairs. MS-DIAL annotation of metabolic features exploited the online library MSMS-Public-Neg-VS15.msp (36,848 records) with the following parameters: retention time tolerance, 100 min; accurate mass tolerance (MS1), 0.01 Da; accurate mass tolerance (MS2), 0.05 Da; identification score cut off, 85%. Putative annotation of differentially expressed metabolites resulted from MS-DIAL screening of the MS1 detected exact HR *m/z* and MS2 fragmentation patterns against multiple online databases (http://prime.psc.riken.jp/compms/msdial/main.html#MSP; Tsugawa *et al.*, 2015). After data-cleaning (blank check, SN > 10, CV QC < 30%), 739 metabolomic variables were retained for further chemometrics. The final dataset was normalized (median normalization, cube-root transformation and Pareto scaling) using MetaboAnalyst (v 5.0; Pang *et al.*, 2021) prior to multivariate statistical analyses.

1. **Collection of *Hpa*-responsive root exudates from soil.**

A population of Arabidopsis Col-0 plants was cultivated in the SBL experimental setup described above but without plastic covers. Two weeks after sowing, half of the pots were inoculated with an *Hpa* spore suspension (50 spores/µl water) or water. Seven days post-inoculation, approximately 65 ml of 5% ethanol was poured on top of the pots and allowed to flow through the soil. Following saturation of the soil, 50 ml of soil effluent was caught in 50-ml Falcon tubes and immediately frozen at -20°C. Samples were lyophilized for further processing.

1. **Collection of *Hpa*-responsive root exudates from agar-solidified medium.**

Arabidopsis seeds were surface-sterilized and sown on agar-solidified 1x Murashige and Skoog medium supplemented with 0.5% sucrose. After 4 days of stratification, plates were transferred to a climate chamber (21°C, 70%, relative humidity, 10 h light/14 h dark, light intensity 100 μmol m^−2^ s^−1^). Twelve-day old seedlings were transplanted to agar-solidified HG medium with 5 seedlings per plate. On the same day, plants were inoculated with *Hpa* spores from a gnotobiotic laboratory-maintained *Hpa* noco2 culture (free of other microbes) by gently touching leaves from the infected plants against leaves of the healthy plants. Healthy plants that remained untreated and plates containing HG agar medium without any plants (empty control) were taken along as well. Plates were placed in sealed trays to ensure high humidity and stored in a climate chamber set at conditions favorable for infection (16°C, 70% relative humidity, 9h light/15h dark, light intensity 100 μmol m^−2^ s^−1^). Seven days post-inoculation, rectangular pieces of agar medium were cut from the area occupied by the root elongation zone of plants that were visibly sporulating. Agar pieces were collected and pooled per plate as one biological replicate. For the empty control, agar samples of similar weight were taken from plates without plants. Samples were snap frozen in liquid nitrogen and stored at -80°C. Samples were lyophilized prior to further processing.

1. **UHPLC-MS/MS analysis of coumarins.**

Lyophilized samples were ground in 1 ml methanol using a tissue lyser (Greiner) with 3.2 mm glass beads for 5 min at 30 Hz and subsequently centrifuged at 9391 *g* for 3 minutes at 5°C. The supernatant was removed and samples were dried by vacuum evaporation. The pellet was then dissolved in 150 µl 25% acetonitrile and samples were finally filtered over a 0.2 µm nylon filter at 7607 *g* for 3 min prior to analysis. Coumarins were separated on an a 1290 Infinity II UHPLC system equipped with an InfinityLab Poroshell 120 PFP, 2.1 x 100 (P/N: 695675-408) in combination with a Poroshell 120 PFP, with a 2.1 mm HPLC guard column and a 1.9 µm LC column (P/N: 821725-942; Agilent Technologies) coupled to an Agilent Technologies 6470A Tandem Quadrupole Mass Spectrometer (TQ-MS-MS). For the gradient elution, 10 mM ammonium acetate and 0.1% formic acid (solution A) and acetonitrile with 0.1% formic acid (solution B) was used. Separation of coumarins was achieved using the following linear gradient: From 90% solution A to 80% solution A in 1.2 min, from 80% solution A to 60% solution A in 2.8 min, hold on 60% for 0.25 min, from 60% solution A to 5% solution A in 1.75 min, hold on 5% for 1.75 min, from 5% solution A back to 90% solution A in 0.25 min and finally hold on 90% solution A for 0.75 min.

Coumarins were subsequently analysed using MS with an ESI source in positive ion mode. Scopoletin was identified using the precursor ion of 193 m/z, and product ions 178, 133 and 77.1 m/z, and quantified using the precursor ion of 193 m/z and product ion 178 m/z. Esculetin was identified using the precursor of 179m/z, and the product ions of 123.1, 105.1 and 77 m/z, and quantified using the precursor ion of 179 m/z and product ion 123.1 m/z. Fraxetin was identified using the precursor ion of 209 m/z, and product ions of 194, 149 and 107 m/z, and quantified using the precursor ion of 209 and the product ion of 194. Fragmentation was achieved with a collision energy of 22 eV for scopoletin, 20 eV for esculetin and 22 eV for fraxetin.

The instrument control, MS data acquisition, and processing were carried out with the MassHunter™ software, version 10.0 (Agilent).

1. **Methods S1 References.**

Asai, S., Shirasu, K. and Jones, J. D. G. (2015). *Hyaloperonospora arabidopsidis* (Downey mildew) infection assay in *Arabidopsis*. *Bio-protocol* **5**:e1627.

Baym, M., Kryazhimskiy, S., Lieberman, T. D., Chung, H., Desai, M. M. and Kishony, R. (2015). Inexpensive multiplexed library preparation for megabase-sized genomes. *PLoS One* **10**:e0128036.

Berendsen, R. L., Vismans, G., Yu, K., Song, Y., De Jonge, R., Burgman, W. P., Burmølle, M., Herschend, J., Bakker, P. A. H. M. and Pieterse, C. M. J. (2018). Disease-induced assemblage of a plant-beneficial bacterial consortium. *ISME J* **12**:1496.

Bolyen, E., Rideout, J. R., Dillon, M. R., Bokulich, N. A., Abnet, C. C., Al-Ghalith, G. A., Alexander, H., Alm, E. J., Arumugam, M., Asnicar, F., Bai, Y., Bisanz, J. E., Bittinger, K., Brejnrod, A., Brislawn, C. J., Brown, C. T., Callahan, B. J., Caraballo-Rodriguez, A. M., Chase, J., Cope, E. K., Da Silva, R., Diener, C., Dorrestein, P. C., Douglas, G. M., Durall, D. M., Duvallet, C., Edwardson, C. F., Ernst, M., Estaki, M., Fouquier, J., Gauglitz, J. M., Gibbons, S. M., Gibson, D. L., Gonzalez, A., Gorlick, K., Guo, J., Hillmann, B., Holmes, S., Holste, H., Huttenhower, C., Huttley, G. A., Janssen, S., Jarmusch, A. K., Jiang, L., Kaehler, B. D., Kang, K. B., Keefe, C. R., Keim, P., Kelley, S. T., Knights, D., Koester, I., Kosciolek, T., Kreps, J., Langille, M. G. I., Lee, J., Ley, R., Liu, Y. X., Loftfield, E., Lozupone, C., Maher, M., Marotz, C., Martin, B. D., McDonald, D., McIver, L. J., Melnik, A. V., Metcalf, J. L., Morgan, S. C., Morton, J. T., Naimey, A. T., Navas-Molina, J. A., Nothias, L. F., Orchanian, S. B., Pearson, T., Peoples, S. L., Petras, D., Preuss, M. L., Pruesse, E., Rasmussen, L. B., Rivers, A., Robeson, M. S., 2nd, Rosenthal, P., Segata, N., Shaffer, M., Shiffer, A., Sinha, R., Song, S. J., Spear, J. R., Swafford, A. D., Thompson, L. R., Torres, P. J., Trinh, P., Tripathi, A., Turnbaugh, P. J., Ul-Hasan, S., van der Hooft, J. J. J., Vargas, F., Vazquez-Baeza, Y., Vogtmann, E., von Hippel, M., Walters, W., Wan, Y., Wang, M., Warren, J., Weber, K. C., Williamson, C. H. D., Willis, A. D., Xu, Z. Z., Zaneveld, J. R., Zhang, Y., Zhu, Q., Knight, R. and Caporaso, J. G. (2019). Reproducible, interactive, scalable and extensible microbiome data science using QIIME 2. *Nat Biotechnol* **37**:852-857.

Callahan, B. J., McMurdie, P. J., Rosen, M. J., Han, A. W., Johnson, A. J. and Holmes, S. P. (2016). DADA2: High-resolution sample inference from Illumina amplicon data. *Nat Methods* **13**:581-583.

Cao, H., Glazebrook, J., Clarke, J. D., Volko, S. and Dong, X. (1997). The Arabidopsis NPR1 gene that controls systemic acquired resistance encodes a novel protein containing ankyrin repeats. *Cell* **88**:57-63.

Clarke, K. R. (1993). Non-parametric multivariate analyses of changes in community structure. *Aus J Ecol* **18**:117-143.

De Muinck, E. J., Trosvik, P., Gilfillan, G. D., Hov, J. R. and Sundaram, A. Y. M. (2017). A novel ultra high-throughput 16S rRNA gene amplicon sequencing library preparation method for the Illumina HiSeq platform. *Microbiome* **5**:68.

Ihrmark, K., Bödeker, I. T. M., Cruz-Martinez, K., Friberg, H., Kubartova, A., Schenck, J., Strid, Y., Stenlid, J., Brandström-Durling, M., Clemmensen, K. E. and Lindahl, B. D. (2012). New primers to amplify the fungal ITS2 region – evaluation by 454-sequencing of artificial and natural communities. *FEMS Microbiology Ecology* **82**:666-677.

Illumina. (2013). "16S metagenomic sequencing library preparation." from <https://support.illumina.com/>.

Lin, H. and Peddada, S. D. (2020). Analysis of compositions of microbiomes with bias correction. *Nat Comm* **11**:3514.

Love, M. I., Huber, W. and Anders, S. (2014). Moderated estimation of fold change and dispersion for RNA-seq data with DESeq2. *Genome Biol* **15**:550.

Luna, E., Flandin, A., Cassan, C., Prigent, S., Chevanne, C., Kadiri, C. F., Gibon, Y. and Pétriacq, P. (2020). Metabolomics to exploit the primed immune system of tomato fruit. *Metabolites* **10**:96.

Martin, M. (2011). Cutadapt removes adapter sequences from high-throughput sequencing reads. *EMBnet J* **17**:10-12.

McMurdie, P. J. and Holmes, S. (2013). phyloseq: an R package for reproducible interactive analysis and graphics of microbiome census data. *PLoS One* **8**:e61217.

Oñate-Sánchez, L. and Vicente-Carbajosa, J. (2008). DNA-free RNA isolation protocols for Arabidopsis thaliana, including seeds and siliques. *BMC Res Notes* **1**:1-7.

Pang, Z., Chong, J., Zhou, G., de Lima Morais, D. A., Chang, L., Barrette, M., Gauthier, C., Jacques, P.-É., Li, S. and Xia, J. (2021). MetaboAnalyst 5.0: narrowing the gap between raw spectra and functional insights. *Nucleic Acids Res* **49**:W388-W396.

Quast, C., Pruesse, E., Yilmaz, P., Gerken, J., Schweer, T., Yarza, P., Peplies, J. and Glockner, F. O. (2013). The SILVA ribosomal RNA gene database project: improved data processing and web-based tools. *Nucleic Acids Res* **41**:D590-596.

Rognes, T., Flouri, T., Nichols, B., Quince, C. and Mahe, F. (2016). VSEARCH: a versatile open source tool for metagenomics. *PeerJ* **4**:e2584.

Schmid, N. B., Giehl, R. F., Döll, S., Mock, H.-P., Strehmel, N., Scheel, D., Kong, X., Hider, R. C. and von Wirén, N. (2014). Feruloyl-CoA 6′-Hydroxylase1-dependent coumarins mediate iron acquisition from alkaline substrates in Arabidopsis. *Plant Physiol* **164**:160-172.

Stringlis, I. A., Yu, K., Feussner, K., de Jonge, R., Van Bentum, S., Van Verk, M. C., Berendsen, R. L., Bakker, P. A. H. M., Feussner, I. and Pieterse, C. M. J. (2018). MYB72-dependent coumarin exudation shapes root microbiome assembly to promote plant health. *Proc Natl Acad Sci U S A* **115**:E5213-E5222.

Tsugawa, H., Cajka, T., Kind, T., Ma, Y., Higgins, B., Ikeda, K., Kanazawa, M., VanderGheynst, J., Fiehn, O. and Arita, M. (2015). MS-DIAL: data-independent MS/MS deconvolution for comprehensive metabolome analysis. *Nat Methods* **12**:523-526.

Van der Ent, S., Verhagen, B. W., Van Doorn, R., Bakker, D., Verlaan, M. G., Pel, M. J. C., Joosten, R. G., Proveniers, M. C., Van Loon, L. C., Ton, J. and Pieterse, C. M. J. (2008). MYB72 is required in early signaling steps of rhizobacteria-induced systemic resistance in Arabidopsis. *Plant Physiol* **146**:1293-1304.

Vismans, G., Spooren, J., Pieterse, C. M. J., Bakker, P. A. H. M. and Berendsen, R. L. (2021). Soil-borne legacies of disease in *Arabidopsis thaliana*. The Plant Microbiome: Methods and Protocols. L. C. Carvalhais and P. G. Dennis, Springer**:** 209-218.

Wickham, H. (2016). ggplot2: elegant graphics for data analysis, Springer.

Wildermuth, M. C., Dewdney, J., Wu, G. and Ausubel, F. M. (2001). Isochorismate synthase is required to synthesize salicylic acid for plant defence. *Nature* **414**:562-565.

Yilmaz, P., Parfrey, L. W., Yarza, P., Gerken, J., Pruesse, E., Quast, C., Schweer, T., Peplies, J., Ludwig, W. and Glockner, F. O. (2014). The SILVA and "All-species Living Tree Project (LTP)" taxonomic frameworks. *Nucleic Acids Res* **42**:D643-648.

Zamioudis, C., Hanson, J. and Pieterse, C. M. J. (2014). Beta-Glucosidase BGLU42 is a MYB72-dependent key regulator of rhizobacteria-induced systemic resistance and modulates iron deficiency responses in Arabidopsis roots. *New Phytol* **204**:368-379.
